# Simulating the Cortical Microcircuit Significantly Faster Than Real Time on the IBM INC-3000 Neural Supercomputer

**DOI:** 10.3389/fnins.2021.728460

**Published:** 2022-01-20

**Authors:** Arne Heittmann, Georgia Psychou, Guido Trensch, Charles E. Cox, Winfried W. Wilcke, Markus Diesmann, Tobias G. Noll

**Affiliations:** ^1^JARA-Institute Green IT (PGI-10), Jülich Research Centre, Jülich, Germany; ^2^Simulation and Data Laboratory Neuroscience, Jülich Supercomputing Centre, Institute for Advanced Simulation, Jülich Research Centre, Jülich, Germany; ^3^IBM Research Division, Almaden Research Center, San Jose, CA, United States; ^4^Institute of Neuroscience and Medicine (INM-6), Institute for Advanced Simulation (IAS-6), and JARA Institute Brain Structure-Function Relationships (INM-10), Jülich Research Centre, Jülich, Germany; ^5^Department of Physics, Faculty 1, RWTH Aachen University, Aachen, Germany; ^6^Department of Psychiatry, Psychotherapy and Psychosomatics, School of Medicine, RWTH Aachen University, Aachen, Germany

**Keywords:** reconfigurable computing, neuromorphic computing, parallel computing, FPGA cluster, spiking neural networks, performance benchmarking, procedural connectivity

## Abstract

This article employs the new IBM INC-3000 prototype FPGA-based neural supercomputer to implement a widely used model of the cortical microcircuit. With approximately 80,000 neurons and 300 Million synapses this model has become a benchmark network for comparing simulation architectures with regard to performance. To the best of our knowledge, the achieved speed-up factor is 2.4 times larger than the highest speed-up factor reported in the literature and four times larger than biological real time demonstrating the potential of FPGA systems for neural modeling. The work was performed at Jülich Research Centre in Germany and the INC-3000 was built at the IBM Almaden Research Center in San Jose, CA, United States. For the simulation of the microcircuit only the programmable logic part of the FPGA nodes are used. All arithmetic is implemented with single-floating point precision. The original microcircuit network with linear LIF neurons and current-based exponential-decay-, alpha-function- as well as beta-function-shaped synapses was simulated using exact exponential integration as ODE solver method. In order to demonstrate the flexibility of the approach, additionally networks with non-linear neuron models (AdEx, Izhikevich) and conductance-based synapses were simulated, applying Runge–Kutta and Parker–Sochacki solver methods. In all cases, the simulation-time speed-up factor did not decrease by more than a very few percent. It finally turns out that the speed-up factor is essentially limited by the latency of the INC-3000 communication system.

## Introduction

In the last decades, significant progress has been made in theoretical and experimental neuroscience giving rise to a tremendous body of available knowledge about biological neural networks (Sejnowski et al., 2014). While the brain dynamics can be resolved from fractions of milliseconds (e.g., the ion-channel dynamics of the cell membrane) to years (long-term learning and brain development), the spatial structure of the brain has been examined from the level of synapses and multi-compartmental dendrites up to multi-area networks of interconnected brain areas featuring millions of neurons and billions of synapses.

Neuroscience researchers improve the understanding of the brain by the systematic elaboration of advanced brain models. These models concentrate on the functional architecture [e.g., Waterloo’s Semantic Pointer Architecture Unified Network, Spaun (Eliasmith et al., 2012)] or on the detailed interplay between brain structure and activity (e.g., models of the cortical microcircuit (Haeusler and Maass, 2007; Potjans and Diesmann, 2014; Markram et al., 2015) and a multi-area model (Schmidt et al., 2018) of the vision related areas of the macaque monkey).

The creation and improvement of these networks currently gets more and more supported by sophisticated generic scientific development environments (Einevoll et al., 2019) such as NEST (Gewaltig and Diesmann, 2007), Nengo (Bekolay et al., 2014), and neuCube (Kasabov, 2014) aiming at multimodal model evaluation by means of simulation – typically carried out on traditional digital high-performance computers (HPC), dedicated compute engines such as Manchester’s SpiNNaker (Furber et al., 2014), Heidelberg’s BrainScaleS (Schemmel et al., 2010), or commercially available general purpose GPU-based machines (Knight and Nowotny, 2018).

At the resolution of nerve cells, network instantiation and the execution of simulation tasks on state-of-the-art supercomputers are by far too slow, especially for the study of plasticity and learning in brain-scale networks. Today, supercomputers reach a simulation speed equivalent to biological real time for the cortical microcircuit (representing 0.0001% of the human brain), and the simulation of advanced multi-area brain models is slowed down by orders of magnitude. Here, the elaboration of dedicated accelerator circuits being either attached to existing high performance compute systems or stand-alone solutions are highly demanded in order to overcome the persistent challenges in speeding up the simulation of biological neural networks.

**FIGURE 1:**
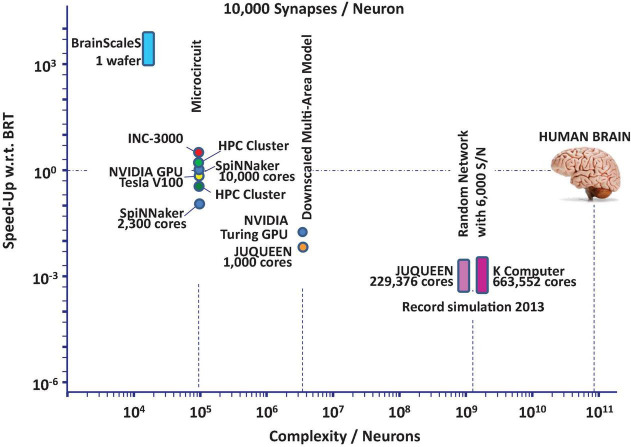
State-of-the-art performance trends based on data found in RIKEN BSI (2013), Schmitt et al. (2017), Knight and Nowotny (2018, 2021), Kurth et al. (2020), Rhodes et al. (2020), van Albada et al. (2021) for neuroscience simulation platforms: speed-up in units of biological realtime (BRT) vs. network complexity in units of neurons assuming essentially random wiring.

The current state-of-the-art in neuroscience simulation is illustrated in Figure 1. The figure shows that network models of significant components of the mammalian brain can be simulated today with severe restrictions regarding the simulation time.

At present these performance limitations can be attributed to significant latencies in available communication technologies used to cope with the required interconnect at full (i.e., biologically meaningful) synaptic connection density. Furthermore, with the incorporation of in-depth neuron dynamics and realistic synaptic rules also critical-path induced latencies of the numerics contribute to the deterioration of the performance.

The aim of the current investigation is to overcome fundamental limitations of present neuromorphic technology. Predominantly, these are the inability to represent neuronal networks at full density, the long times required for network generation, and the difficulties to reach a processing speed significantly faster than real time at a moderate level of flexibility in neuronal and synaptic dynamics. The latter is relevant as processes of system-level learning and development unfold over minutes and hours of biological time.

The next-generation neuroscience simulation platform has to be designed such that the *common case* of relevant neuroscience brain models is executed in the most efficient way, the required design time and costs stay within an acceptable range, and system integration density assures that inter-node communication can be carried out with ultra-low-latency. Particular attention will be paid on the designed-in flexibility of the dedicated accelerator circuits, which will be restricted and optimized to the NC-specific application domain in order to achieve significant speed up. It is to be expected that these challenges cannot be solved without a sophisticated design-space exploration with regard to the architectural organization of a digital simulation platform. Here, the IBM INC platform (Narayanan et al., 2020) serves as a means to implement prototypical circuits and architectures for the envisioned next-generation neuroscience simulation platform (Configurable Spiking Neural Network Simulations, CsNNs. All used acronyms and symbols are explained in Supplementary Data Sheet 1) in order to identify bottlenecks and critical algorithmic sections in the simulation flow.

## Materials and Methods

### The IBM INC-3000 Neural Supercomputer

#### Motivation for the Development of the IBM Neural Supercomputer (INC)

The IBM Neural Supercomputer (Narayanan et al., 2020) originated as part of the IBM General Artificial Intelligence (GAI) project (aka Machine Intelligence Project) at IBM Research in Almaden, California. This project aims to develop an architectural model of the neo-cortex which is based on key elements of what neuroscience has learned about the structure and functioning of the mammalian brain. This is a Goldilocks problem – we want to utilize just enough of this biological information to enable GAI, but not slavishly follow all the known details of the brain’s architecture. As an analogy – if the brain were a tree, then we want to capture concepts like roots, trunk, branches, leaves, but not the detailed location of each leaf.

The details of this approach are out of scope for this article, but among the key ideas are that learning in the brain is not based on adjusting weights in a very densely connected network – as it is done in conventional machine learning – but forming new connections in a sparsely (<1%) connected network of neurons. As the project has demonstrated, this leads to the fundamental capability of such a plastic network to extract invariant (stable) representations from temporal varying sensory input without supervision. This has been demonstrated in a recent article by the IBM Almaden team (Scott et al., 2019).

The traditional machine learning model of dense matrix-vector operations maps nicely on GPUs but GPUs are not good platforms for the sparse and dynamically changing (plastic) topology of realistic brain models. A large traditional message-passing supercomputer would also be a good platform for the GAI model, but IBM does not have internal access to big supercomputers. So we decided to build our own platform based on large FPGA nodes. Since GAI algorithms are very much under development, the extreme flexibility provided by FPGA systems was a deciding factor to go this route. Also, if an application lends itself to deep pipelining, FPGAs can provide multiple order of magnitude performance improvements over traditional CPU systems of comparable cost.

We decided to build a large (16–1,296 nodes) system with special focus on good connectivity between the nodes. It is organized as a massively parallel 3D mesh of reconfigurable computing nodes, where each node consist of a large Xilinx FPGA including two ARM cores, external DRAM, a programmable memory and twelve high speed serial transmitters.

**FIGURE 2:**
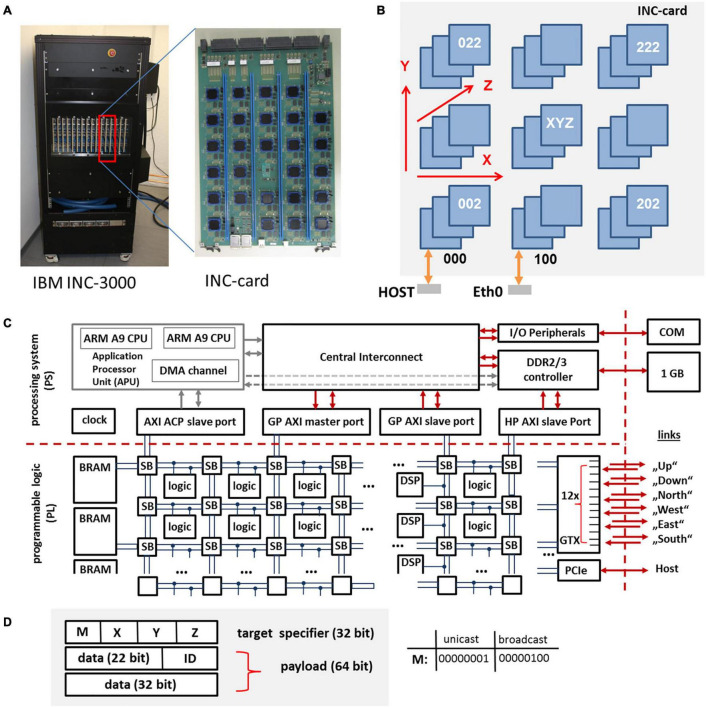
INC-3000 system featuring 16 INC cards **(A)**, each card **(B)** is equipped with 27 FPGAs. The whole INC-system represents a 12 × 12 × 3 3D-node mesh. The architecture of the Xilinx XC Z7045 SoC is shown in **(C)**. The programmable logic (PL) is based on a fabric of configurable logic blocks, DSPs, and block RAMs (BRAM). The packet format (96 Bit) is shown in **(D)**. A packet consists of a 32-bit target specifier and a 64 bit payload. The triplet (*X, Y, Z*) specifies the logical coordinate of the target node (unicast mode). The ID-field specifies the packet type.

The basic unit of the INC is a large 48 layer custom card with 27 nodes connected on-card as a 3 × 3 × 3 cube, cf. Figures 2A,B. An INC system contains 1 cage (INC-3000) or 3 cages (INC-9000). Each cage contains 16 cards. Two INC-3000 systems have been built so far, one for IBM Almaden and the other for Jülich Research Centre. The INC-3000 systems contain 16 × 27 or 432 nodes.

The INC-3000 systems have already been used – in addition to the major application discussed in this present article – for demonstrating the formation of invariant representations as discussed above and for very fast AI learning of video games based on genetic algorithms (“neuroevolution”) (Asseman et al., 2020).

For this particular project, the INC machine serves as a prototyping platform. Based on the results obtained from prototypical circuit realizations the aim is to elaborate an optimized dedicated hardware platform based on customized accelerator-circuits. In particular, the question regarding a most appropriate node topology for the realization of architectures suitable for the simulation of spiking neural networks (e.g., based on a tree-like interconnect structure with ultra-low latency packet transmission) is very important.

#### The INC-3000 Compute Node

Each of the Xilinx XC Z7045 System on a Chip (SoCs) (Xilinx, 2018b) contains ∼218k programmable 6-input lookup tables (LUTS – configurable logic), ∼437k flip-flops, 900 dedicated customizable DSP blocks for the implementation of arithmetic functions, 19.2 Mbit Block-RAM (BRAM, realized as static random access memory), and 16 serial GTX transceivers with a maximum data rate of 12.5 Gb/s in each direction. For INC-3000, 12 GTX transceivers are used in order to establish the bidirectional interconnect architecture, cf. Figure 2C. DSP blocks and BRAMs are organized in slices and can be accessed *via* the interconnect fabric. On an INC-3000 compute node, circuits are subdivided into two clock domains. While the logic for the communication system is clocked with a clock frequency of 100 MHz, the application logic is clocked with *f*_clk_ = 150 MHz. Both domains are interfaced *via* dedicated FIFO circuits running at 250 MHz.

In addition to the logic fabric, an ARM-A9-subsystem is provided on each SoC, comprising two independent ARM cores running at 1 GHz, various peripherals, an AXI bus system, and an interface to an external 1 GB DDR memory system per node. The external memory system can be accessed by the fabric logic (without using the ARM cores) *via* dedicated AXI-channels and can be used to store configuration data, simulation results, as well as state variables. Currently, for the simulation of spiking neural networks on INC-3000 solely the programmable logic (PL) resources are used.

#### The INC-3000 Communication System

In the following, the SoC devices are the key components of *nodes*. Each node includes the complete router logic necessary to post, relay, and deliver data packets. On INC-3000, communication is realized *via* a three-dimensional mesh of locally interconnected nodes. Individual nodes can be addressed *via* an unique identifier which specifies the absolute node location in terms of its coordinates (*X, Y, Z*) in the mesh. Communication channels are physically realized to the nearest neighbors (“up,” “down,” “north,” “south,” “east,” “west”) of a node *via* gigabit GTX transceivers. There are also express connections to non-nearest neighbors to decrease hop count, for a total of twelve links emanating from each node. The smallest amount of information which can be transmitted on INC-3000 is given by a 64 bit payload and a 32 bit target node specifier which contains information about the target node which receives the packet, cf. Figure 2D. Node (0, 0, 0) provides a separate PCI-express interface (PCIe) to a host system which is used to configure, monitor, and control the INC-3000 system. There are two PCIe connections from each card to an external host computer.

Packets can be sent in two distinct modes: One mode directly and uniquely addresses a single target node (unicast routing) *via* specification of the target coordinates (*X, Y, Z*). Using the second mode, a packet is submitted in an unspecific way to all nodes located in the INC-3000 system (broadcast routing). The routing method applies deterministic *dimension-order-routing* in order to direct a packet to its targets.

Nodes fall apart into two classes, depending on their purpose in the system architecture: So-called *compute nodes* (CNs) actively participate in the solution of the neuro-synaptic dynamic equations, while so-called *relay nodes* (RNs) take over special functions for the management and organization of communication. Any node in the INC-3000 system can be configured to act as CN, RN, or both. For the current application, a single node is configured as an RN which is subsequently called *master node* (MN). The MN manages the node synchronization and distributes spike events to all CNs in the system.

### Spiking Neural Networks

#### Spiking Integrate-and-Fire Neuron Models

Almost all biophysically grounded spiking neuron models are based on ordinary differential equations and systems of it. Depending on the required level of details it is possible to break down the neuro-synaptic dynamics to the dynamics of individual ion channels which results, e.g., in Hodgkin-Huxley-like coupled equations systems. It turns out to be computational expensive to obtain a solution of these equation systems. To be able to simulate networks of significant size, simplified models with focus on the subthreshold dynamics of the neuron’s membrane voltage are used, with only few coupled state variables, (e.g., Izhikevich, 2003; Brette and Gerstner, 2005; Yamauchi et al., 2011). The simplest model of the membrane dynamics is due to Lapicque (1907) and described by a single dynamic equation, the so-called leaky integrate-and-fire (LIF) model. Here, the dynamics of the membrane is given by,


(1) $$C_{m}\cdot \frac{{\mathrm{dV}}_{q}}{\mathrm{dt}}=-g_{m}\cdot \left(V_{q}-E_{L}\right)+I_{m,q}+I_{\mathrm{ext}}.$$

In (1) *C*_*m*_, *V*_*q*_, *E*_*L*_, *g*_*m*_, *I_*m,q*,_ I_*ext*_*, denote the membrane capacitance, the membrane potential of neuron *q*, the resting potential, the membrane conductance, the aggregated synaptic membrane current, and an external current, respectively. The aggregated synaptic current represents activity that has arrived at the neuron from the remainder of the network. Realistic neurophysical models incorporate at least two types of synapses: excitatory synapses (e) and inhibitory synapses (i). Then, the current *I*_*m,q*_ splits up into two types of synaptical currents: an excitatory *I*_*m,q,e*_ and an inhibitory *I*_*m,q,i*_ one. For the cortical microcircuit model, the concept of *lumped synapses* (Rotter and Diesmann, 1999) is used, which essentially implies that dynamic equations have to be considered for specific *synaptic types* instead of synaptic instances, only. The following equation describes synapses with exponential decay and current-based coupling (CUBA):


(2) $$\tau_{s,x}\cdot \frac{dI_{m,q,x}}{dt}=-I_{m,q,x}+I_{S,q,x}\;,x\in \left\{e,i\right\}.$$

In (2), τ_*s,e*_ (*τ_*s,i*_*), *I_*m,q,e*_ (I_*m,q,i*_)*, *I*_*S,q,e*_ (*I*_*S,q,i*_) denote the synaptic time constant of excitatory (inhibitory) synapses, the aggregated postsynaptic membrane current, and the aggregated excitatory (inhibitory) synaptic input, respectively. With (2), the overall membrane current is given by the sum *I*_*m,q*_ *= I_*m,q,e*_+I_*m,q,i*_*. Finally, the so-called *synaptic input* is derived from the incoming (presynaptic) spike train *S*_*P*_,


(3) $$I_{S,q,x}\left(t\right)=\sum_{p\in B_{x}}J_{qp}\cdot S_{p}\left(t-D_{p,q}\right)\;,x\in \left\{e,i\right\}.$$

In (3) *p, J_*qp*,_ D_*p,q*,_ t, S_*P*_* describe an index of a presynaptic neuron, a synaptic strength, a transmission delay, the time *t*, and a spike train *S*_*p*_ = Σ_*k*_δ(*t-t*_*k,p*_), respectively. The set *B_*e*_ (B_*i*_)* represents the set of excitatory (inhibitory) neurons: Excitatory neurons drive excitatory synapses, while inhibitory neurons drive inhibitory synapses.

#### The Microcircuit Model

The evolutionary youngest part of the mammalian brain is the neocortex, cortex for short, a sheet of roughly 1 mm thickness covering the surface of the brain. This structure has increased in volume by three orders of magnitude from mouse to man while its local structure remained essentially unchanged. Furthermore, the structure looks very similar in parts of the cortex responsible for the processing of visual information, auditory information, or the planning of motor tasks. This dual universality gives neuroscientists hope that fundamental computational principles can be conserved by evolution and reused again and again. The microcircuit model (Potjans and Diesmann, 2014) represents the network below a patch of ∼1 mm^2^ surface of the early mammalian sensory cortex. The model is organized into four layers (L2/3, L4, L5, and L6), each incorporating two populations (an excitatory population and an inhibitory population) what results in a total number of eight separate populations (L2/3E, L2/3I, L4E, L4I, L5E, L5I, L6E, and L6I). The synaptic connections are defined by population-specific connection probabilities of randomly distributed neuron-to-neuron connections. Synaptic strengths and synaptic transmission delays are modeled by normally distributed random numbers. Compared to the Brunel model (cf. Brunel, 2000 and section “Simple Example: Random Networks” in the Supplementary Material), the cortical microcircuit features a more realistic interconnect model of a multiplicity of populations. For the cortical microcircuit the number of involved neurons is sufficiently high to produce a significant amount of internal synaptic connections making the dynamic properties of the network more dependent on the network itself than on the external input. About 50% of the total connections originate from the model itself. However, in order to mimic the remaining 50% of (excitatory) synaptic input from adjacent cortex and other cortical areas, individual neurons receive Poisson-distributed independent spikes with population specific rates. The neuroscientific relevance of the model is that it constitutes the smallest network in which two characteristic parameters can simultaneously be realized: the local connection probability of 0.1 and in the order of 10,000 synapses per neuron. The volume also covers the space in which a neuron establishes most of its local synapses. In this respect the model constitutes a unit cell of the cortical network. The ability to simulate networks of this size was a breakthrough because in smaller models either the connection probability needs to be increased or the synaptic strength needs to be increased to achieve realistic levels of activity. This raised the question on the appropriate procedure of down scaling and whether results obtained for downscaled models hold for the true size. Indeed, (van Albada et al., 2015) showed that the mean of neuronal spiking activity can perfectly be preserved in downscaling, but that there are severe limits already for the second order statistics. Synaptic plasticity, however, directly depends on the correlation structure of the network. In addition, the correlations between neurons drive the fluctuations of neuronal activity observed on the network level by measures like the local-field potential (LFP). As the microcircuit model captures all the synapses between the neurons, from this size on, memory consumption grows only linearly with network size. Because of the falloff of connection probability with distance, larger networks are necessarily less densely connected and should therefore be easier to simulate.

The microcircuit model applies the LIF model for neurons and synaptic currents with exponential decay. The complete parameterization including the network architecture is shown in the Supplementary Tables 4A–H and Supplementary Figure 8.

### CsNNs on INC-3000

#### The CsNNs Compute Node and Simulation Flow

**FIGURE 3:**
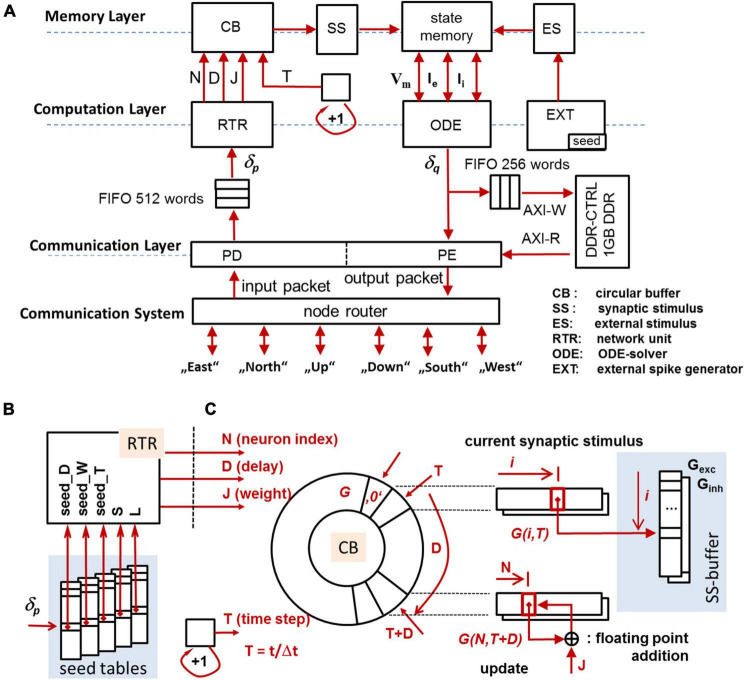
Overview of the CN-architecture **(A)**, seed tables for the MTBRNG-based RTR unit **(B)**, addressing scheme for the circular buffer **(C)**. The circular buffer preserves the forcing terms of synaptic activity to be effective in a future time step.

Figure 3A gives a general overview of the components integrated in a compute node (CN, see section “CsNNs Microarchitecture and Logic Design”). The architecture is divided into (i) a memory layer retaining state variables, input, and stimuli, (ii) a computation layer which operates on the memory to transfer and update state variables, and (iii) a communication layer responsible for posting and receiving spikes to and from the communication system (CS), configuration as well as monitoring. The modules in the computational block are dedicated to execute tasks related to solving eqs. (1–3) and managing data involved herein.

The hardware resources of the *k*-th CN are capable of simulating the dynamics of a set of neurons *W*_*k*_ including the associated synaptic connections. With respect to the synaptic connections, *W*_*k*_ represents a set of postsynaptic neurons. While the size of the set *W*_*k*_ is limited to |*W_*k*_| ≤ 256*, a network of considerable size as a whole is mapped to a number of nodes. Neurons hosted by a particular CN always belong to the same population, and the respective subnetwork hosted on a CN is subsequently called a *micro-cluster*. At this, the procedure of mapping neurons to CNs guarantees a consistent distribution of neurons to CNs in the particular sense that the respective sizes | *W_*k*_|* are of almost equal magnitude.

The simulation sequence is based on a time-driven simulation loop (Brette et al., 2007). The biological time *t* is subdivided into discrete time-steps *t*_*i*_ (also called *algorithmic* time) while a fixed time step size of *t*_*i*_ – *t*_*i*–1_ = Δ*t* = 0.1 ms is assumed. As the simulation is carried out on globally asynchronous CNs, a particular synchronization method for the node is applied on INC-3000 which is based on the exchange of both barrier messages and synchronizations messages. The node synchronization continuously synchronizes the algorithmic time on the CNs.

The operation of a CN is initiated once a sync-message is received by the packet decoder (PD) of the communication layer. Sync messages will be typically provided by the MN which, in brief, indicate that a new simulation time step is ready for computation. The first step of the computation is to transfer the currently valid aggregated synaptic input (3) to the SS-buffer [Synaptic inputS (SS)] and to prepare the ES-buffer [External Stimuli (ES)] with the external stimuli. After the buffers are updated, the ODE-unit [Ordinary Differential Equation (ODE) solver] updates the state memory based on the solution of the dynamic eqs. (1, 2) under the consideration of (3). All involved arithmetic operations are carried out with single-float precision. In the current implementation, simple LIF neurons and lumped synapses featuring exponential decay with CUBA current contribution are implemented whose solution of (1–3) is based on the method of exact integration (Rotter and Diesmann, 1999). However, the implementation of models with elaborated biological plausibility requiring sophisticated ODE-solver architectures will be discussed in section “Performance for Alternative Neuron Models and Ordinary Differential Equation Solvers.”

After the update of the states, the membrane voltage *V*_*q*_ is compared with a given threshold voltage in order to trigger spikes. In the case that *V*_*q*_ exceeds the threshold, the global source-ID of the spiking neurons is looked-up (using an index-translation-table), and a subsequent transfer of the source-ID to the packet encoder (PE) is carried out. Spikes will be distributed among CNs *via* source-encoded AER-packets [Address Event Representation (AER)]. In addition, spike events submitted to the PE are recorded in the DRAM. Presynaptic neurons carry out effect on neurons from the set *W*_*k*_ by posting spikes to the communication system *via* AER-packets. The node router transmits the spike packet to the MN *via unicast routing* which transmits a source-encoded AER-packet to all CNs *via* an undirected broadcast packet. The AER-packet will be received by the packet decoder (PD) which transfers the enclosed source ID, represented by the unique identifier δ_*p*_, to the input FIFO. The identifier δ_*p*_ is initially blocked in the FIFO and subsequently released when the receiving RTR-unit [local spike RouTeR (RTR)] is able to process δ_*p*_. The RTR-unit picks the source IDs from the input FIFO one-by-one in order to update a circular buffer (CB) which aggregates the synaptic input.

Specifically, the RTR-unit operates the local network of synaptic connections. The RTR-unit holds data structures which describe the full set of synaptic connections regarding the set *W*_*k*_ and for any given δ_*p*_. Thus, the function of the RTR-unit is directly related to eq. (3) and ensures that for any (biological) simulation time step *t*_*k*_ the synaptic input are known for the update (2). While the RTR-unit is driven by spikes submitted by neurons in the neural network, the EXT-unit [EXTernal spike generator (EXT)] creates and handles the *external spikes* (see section “The CsNNs Spike-Distribution, Generation, and Synapse-Parameter Look-Up”).

Each synaptic connection is described by a quadruple *Q = (*δ,*J,D,N):* δ represents the unique identifier of the presynaptic neuron, *J* represents the synaptic strength, *D* represents the synaptic transmission delay, and *N* denotes the unique (local) identifier of the postsynaptic neuron. Given the input δ_*p*_, the RTR-unit retrieves all quadruples *Q*_*i*_ which fulfill δ≡δ_*p*_ and consecutively updates the *circular buffer* CB with regard to (*J,D,N*)_*i*_, cf. Figure 3C. The circular buffer retains all intermediate states derived from (3) for the synaptic input based on the most recently incoming spike trains, synaptic strengths, and synaptic transmission delays. On INC, the CB is capable of preserving prospective synaptic input up to 6.4 ms biological real time (BRT) at the resolution of the time step size Δ*t* = 100 μs.

#### CsNNs Pseudo-Random Number Generators

As will be shown below, CsNNs make extensive use of pseudo-random number generators (PRNGs). Recall that a PRNG is a deterministic algorithm that produces pseudo-random numbers whose distribution is almost indistinguishable from that of a true random number. Typically, the synaptic connectivity, transmission delays, and weights are drawn by a PRNG during the network-construction phase upfront to the simulation of a random neural network (NN). The results are stored in huge connectivity and synapse-parameter tables, which are looked-up in the actual simulation phase. This approach can be very much network-construction-time consuming as well as memory hungry.

In contrast to using connectivity tables, the *on-line computation* of the connections reduces the required amount of memory dramatically (Jahnke et al., 1999). First approaches in this direction were carried out for regular connection schemes which follow (simple) deterministic rules and where regular connections can be computed efficiently (Roth et al., 1995, 1997). This approach, however, can also be applied to irregular (i.e., random) connections. It will be shown in the following that re-creating all these pseudo-random numbers (deterministically) during the simulation again and again can be surprisingly more efficient, as long as highly efficient PRNGs are involved. This is one of the core ideas that lies at the heart of CsNNs. On INC-3000, various table-based random number generators were implemented which play a significant role for the generation of external spikes trains and for the generation of a local interconnect structure in the RTR-unit. Table-based PRNG methods offer the advantage of being flexible with respect to the implementable probability mass function (*pmf*) with restricted support (i.e., with a restricted range of acceptable output values), and require only few table lookups to generate random numbers with a particular *pmf.*

On INC-3000, variants of Walker’s alias method (Walker, 1974, 1977) have been implemented which allows to draw a random variable with almost arbitrary probability distribution *p*_*r*_ at O(1) time. In Walker’s algorithm, a set of pairs (*p_*r*_,r*), *r = 1,…,z* was given whereby the condition *Σ_*k*_p_*k*_ = 1* had to be fulfilled. Aim of the described method was to provide a pseudo random number generator which generates random (integer) numbers *r* whose empirical *pmf* approaches *p*_*r*_ for large sample sequences. In the following, the integer random variable *r* is generally replaced by a continuous substitute *R(r)*. A one-to-one mapping of *R* to *r* allows to approximate distributions of continuous random variables by a discrete *pmf*. Here, continuous intervals of a random variable *R* will be represented by a respective average *R* which in turn is represented by a unique integer number *r*. The associated probability density functions (*pdf*) can be aggregated into a unique bin which results in the specification of *p*_*r*_.

**FIGURE 4:**
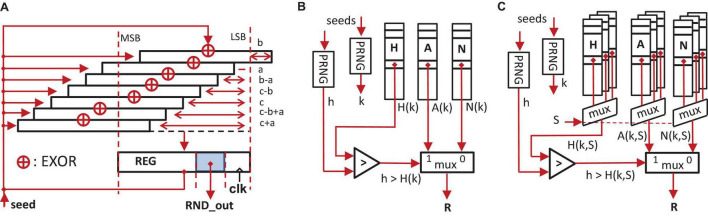
**(A)** Xorshift-based implementation of a simple PRNG (Marsaglia, 2003b). **(B)** TBRNG. The index *k* represents a table address. **(C)** Multidimensional table-based random number generator (MTBRNG). The signal *S* selects a particular set of a (*H,A,N*) table.

In Figure 4B the fundamental architecture of the generator is shown. The method requires three arrays (*H,A*,*N)* and two independent PRNGs generating uniformly distributed RNs. The first PRNG generates an index *0 ≤ k < z* (*z* is the number of table entries), used to select three table locations *H(k), A(k)*, and *N(k)*. The second PRNG generates a RN 0 ≤ *h* < 1 which is then compared against *H(k)*. Dependent on the outcome of the comparison, either *A(k)* or *N(k)* is selected as the output value *R*. In order to simplify the comparison operation, the array *H* uses 32-bit fixed-point representations of scaled probabilities *p*_*k*_ of the *pmf*. Note that the table entries of (*H,A*,*N*) can be calculated offline for given *pmf* in *O(z×log(z))* or better (Vose, 1991). In Figure 4C a variant of a table-based random number generator (TBRNG) is shown which features an additional parameter *S*, which is used to select a particular set of (*H, A*, *N)-*tables. In the following, this kind of TBRNG is called *multidimensional* TBRNG (MTBRNG).

The PRNGs are based on xorshift-generators (Marsaglia, 2003a,b; Vigna, 2014; Blackman and Vigna, 2018), cf. Figure 4A. Xorshift PRNGs are very simple in its structure and provide sufficiently long sequence periods for particular parameterizations. Typically, the update of the internal state of Xorshift-based PRNGs can be finished within a single cycle on the FPGA-logic, e.g., the initiation interval *II* is equal to 1 (see section “CsNNs Microarchitecture and Logic Design” for the definition of *II*).

#### The CsNNs Spike-Distribution, Generation, and Synapse-Parameter Look-Up

As will be shown in the Supplementary Material (see section “Locality Properties”) in case of the microcircuit and a chosen micro-cluster size of 256 neurons, practically every incoming spike needs to be projected to at least one neuron in every micro-cluster. A micro-cluster is defined by the sub-network which emerges from the local set of 256 postsynaptic neurons and the full set of presynaptic neurons defined for the microcircuit. When a spike arrives, based on the global source ID, the CN needs to identify the local target ID and the according synapse parameters have to be looked-up from a table. The first task requires some associative memory functionality, while the second one requires quick reading from a large memory. For the microcircuit, there are 77,169 source IDs (demanding at least 17 address bits). A synapse described by a quadruple *Q = (δ,J,D,N)* requires 32 bits to represent a single float strength *J*, 6 bits to represent a synaptic transmission delay *D* (under the assumption of a maximum synaptic delay of 6.4 ms), and 8 bits to encode a unique (local) identifier *N* for a postsynaptic neuron of a micro-cluster. The costs for the representation of the source ID δ typically can be neglected as data structures can be optimized in such a way that δ is stored (or represented) only once per source neuron. Having an average connection probability of ∼5% (i.e., 50% synaptic input from the network) a typical node has to represent more than ∼0.94M synapses using ∼43 Mbits of synaptic information neglecting additional memory space which is required for organizing data structures. On INC-3000, memory is available both as low-latency BRAM and as high-capacity DRAM (externally attached to every SOC) as well. Because a Z7045 SoC comprises only 19.2 Mbit BRAM (Xilinx, 2018b), it is not feasible to keep a local synaptic connection structure of a sub-network in terms of (*J,D,N*) tuples in the BRAM.

In order to make use of the low-latency BRAM, a new representation of synaptic connectivity is proposed which allows for storing a full sub-network in it in a compressed way. The proposed approach of representing (*J,D,N*) tuples exploits the fact that the connectivity pattern in the microcircuit obeys a random distribution. First, let’s consider a simple Xorshift PRNG (Figure 4A). The PRNG is able to generate a sequence of (almost) uniformly distributed states. This is in particular true if the parameters *a, b*, and *c* are chosen such that the generator produces a sequence of maximum length (Marsaglia, 2003a,b). If the PRNG is deterministically initialized with a seed, the sequence is deterministic as well, but looks random, nevertheless. The initial seed – to some extent – represents the derived sequence in a very memory-efficient way: for long sequences it requires more bits to explicitly store the sequence compared to the option to store the seed only.

The same principle applies to TBRNGs. If a particular seed is used to initialize a TBRNG, the sequence of successive output values is deterministic, and the observable distribution of the output values follows the programmed discrete *pmf*. In that sense, PRNGs are appropriate to generate (pseudo-) random local addresses *N* to postsynaptic neurons, and TBRNGs can be used to generate sequences of pseudo-random synaptic transmission delays *D* as well as pseudo-random synaptic strengths *J*. An incoming spike δ_*p*_ is directly used to address so-called seed-tables (Figures 3A,B). Individual seeds are selected and used for the initialization of the MTBRNGs located in the RTR-logic. Two additional parameters are read out which specify the overall length of the sequence (parameter *L*) and a specifier *S* which is used to select a particular set of (*H,A,N)-*tables of the MTBRNGs. The parameter *S* represents the population membership of the spike δ_*p*_. By addressing a particular set of (*H,A,N)-*tables, networks with population-specific statistical properties can be maintained. The parameter *L* is (roughly) equivalent to the number of synapses on a given (local) axon and specifies the sequence length. A detailed strategy how to define the parameter of the (*H,A,N*)-tables is explained in the Supplementary Material (see section “Twin Numbers, Autapses, and Multapses”).

In addition to spikes generated from the network, so-called *external spike trains* contribute to the excitatory synaptic input in (2). However, unlike in (3) where spikes can be delayed by multiple time steps (which requires the CB to handle delayed spikes), the synaptic strength is subject to variation, and the synaptic multiplicity is random, the external spike train is modeled much simpler: The external spike train acts purely excitatory, the external neurons fire independently with identical firing rates, and the synaptic strengths are identical for all connections (all given a particular target neuron *q*). Eq. (3) gets additively extended by the term,


(4) $$\begin{array}{c} I_{S,q,e}\left(t\right)=J_{q}\cdot \sum_{p\in B_{x}}\left(\sum_{k:\left(t-t_{k,p}\right)<\Delta t}\delta \left(t-t_{k,p}\right)\right)\\ \;\approx J_{q}\cdot M_{q}\cdot \sum_{k:\left(t-t_{k,p}\right)<\Delta t}\delta \left(t-t_{k,p}\right) \end{array}$$

In (4) *J*_*q*_ and *M*_*q*_ describe the common synaptic strength of the external input and the fan-in from other cortical areas to neuron *q*. For a given average spike rate *λ_*q*_* [spikes/s] and simulation step size Δ*t*, the term Σ_p_ Σ_k_δ(*t-t*_*k,p*_) obeys a *Poisson* (*M_*q*_×*Δ*t*×λ_*q*_) distribution. Based on the given *pmf*, a TBRNG can be used to generate the aggregated external spike input followed by a multiplication (4). Equivalently, the *pmf* of *I*_*S,q,e*_ related to the external spike train can be gathered in order to train the (*H,A,N*)-tables of the TBRNG. One advantage of the latter case is that the multiplication operation in (4) can be completely avoided. The complete statistics of the target variable can be stored in the tables.

#### The CsNNs Communication and Synchronization Concept

The communication architecture has three objectives: the synchronization of the compute nodes, the transmission of spike packets, and setup, monitoring, and control of the INC-3000 system. In CsNNs on INC-3000, a time-driven simulation approach has been established which requires a particular synchronization strategy. The INC-3000 system can be characterized (as almost all massively parallel computing systems) as operating globally asynchronous and locally synchronous (GALS): Individual nodes are driven by unsynchronized clocks and finish their computations for given time steps at different wall-clock times due to different temporal load profiles (e.g., caused by the number of synapses per incoming spike, etc.). In order to minimize possible spike losses, the algorithmic simulation time steps are synchronized over the whole GALS system by “barrier messaging,” (cf. e.g., Culler et al., 1998; Parhami, 2002).

On INC-3000, the concept of *barrier messages* is used to synchronize nodes: At some wall-clock time, within an algorithmic time step, the ODE solver finishes its operation after all state variables have been updated, and the detected spikes are delivered to the communication system. At this point, the CN can proceed to the next time step after all spikes from the other nodes are received, the synaptic inputs are known, and the circular buffer has been updated.

**FIGURE 5:**
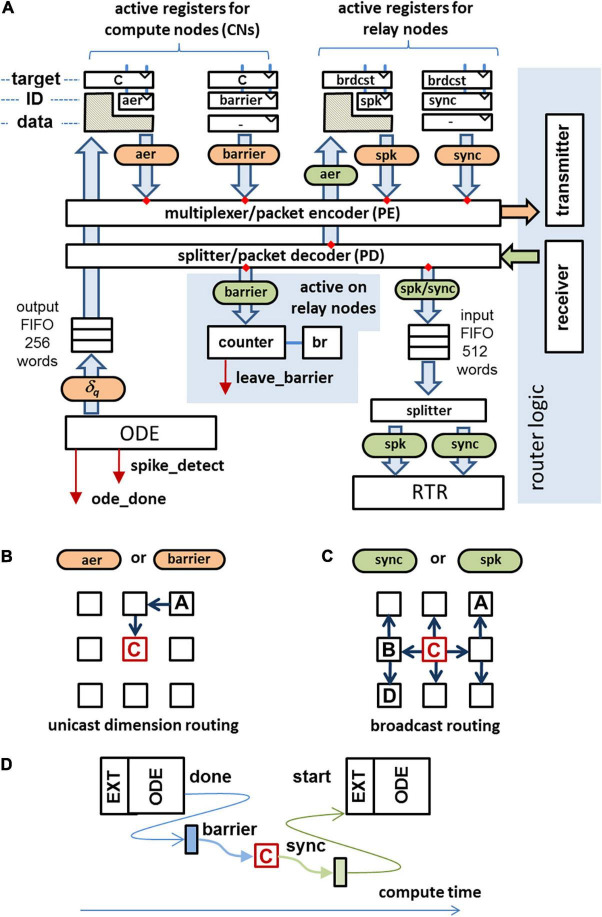
**(A)** Data paths for communication in compute/master nodes. Received packets are marked in green, packets to send are colored in orange. The data fields of crosshatched regions will be replaced by the data field of the incoming packet. **(B)** *Unicast routing* used for the transmission of barrier messages and AER messages. **(C)** *Broadcast routing* used for the transmission of sync packets and spike packets. **(D)** After finishing the ODE processing a barrier message is sent to the master node. As soon as the barrier messages from all active CNs are received, the master node generates a sync message, allowing the CNs to proceed to the next algorithmic time step.

On INC-3000, a dedicated *master node* (configured as a *RN*) is used to coordinate the compute node synchronization. In the case that a particular CN has finished its computations for the current algorithmic time step, it sends a *barrier-message* to the master node, cf. Figure 5D. Once the barrier messages from all active CNs are received by the master node, it broadcasts a so-called *sync-message* to all nodes which allows them to proceed with the next algorithmic time step. The cost in terms of latency to establish a barrier-based synchronization method has been experimentally evaluated. A cluster of nodes of the INC-3000 system was selected imitating active CNs, along with a MN, spatially placed in the center of the cluster. This placement minimizes the worst-case synchronization latency. All CNs were forced to immediately re-deliver barrier packets when a sync-message was received. This results in a well-defined time interval between adjacent sync messages, the *synchronization latency.*

**FIGURE 6:**
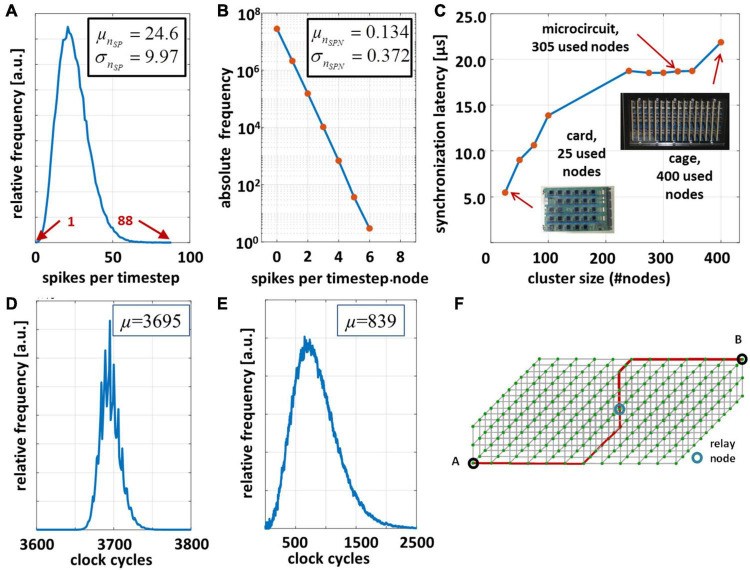
Performance evaluation of the microcircuit cortical model on the INC-3000 system for *t*_BRT_ = 15 min. and simulation time step Δ*t* = 100 μs. **(A)** Distribution of the experimentally observed spike density [measured in (spikes/timestep)]. **(B)** Distribution of generated spikes per node per time step. **(C)** Synchronization latency dependent on the cluster size of active nodes. **(D)** Distribution of elapsed clock cycles between logical time steps. **(E)** Distribution of elapsed clock cycles to unroll the node-local connection structure *via* the RTR-unit. **(F)** 3D-node mesh. The nodes A and B are separated with maximum Manhattan-distance.

The results are shown in Figure 6C. The synchronization latency depends on the cluster size, the node-to-node-latency, and in particular on the Manhattan-distance between the MN and the CNs located at the edges of the cluster (Figure 6F). The maximum round-trip delay time turns out to be 18 μs for a cluster containing 305 CNs. A one-hop latency (first-bit-to-first-bit) of ∼1 μs was measured for the transmission of a single packet. For larger cluster sizes, the cluster diameter grows smoothly with the cluster size. The synchronization latency gives the minimum amount of wall-clock time to process an algorithmic time step and hence yields a lower bound to the achievable speedup factor. Here, the maximum achievable speedup factor *G* approaches 5.6 assuming a simulation time step size of Δ*t* = 100 μs.

Whenever a synchronization message is received by a CN, it can leave the barrier and proceed to process the next algorithmic time step. However, there are two additional conditions to be fulfilled: (i) All spikes submitted by other nodes were received, and (ii) all received spikes were processed and the circular buffer is updated, accordingly. Condition (ii) is sufficiently fulfilled when the input FIFO (cf. Figure 5A) is empty and the RTR-unit is in the IDLE-state. However, condition (i) does not depend on any local state of a given node and hence needs to be fulfilled *implicitly* when a synchronization message is received. While the generation of a sync message by the MN guarantees that no node has outstanding spikes to be submitted, it does not guarantee that the last spike has been received *prior* to the reception of the synchronization message.

A sufficient condition to fulfill (i) is to make sure that the AER packets take the same (deterministic) route like the one of the barrier packet and the spike packets take the same route as the one the sync packet takes. For that reason it is useful to have the MN not only to broadcast the sync packets but also to broadcast the spike packets (cf. Figure 5C). CNs send spikes as *AER-packets* to the MN using a unicast routing protocol, cf. Figure 5B. On the MN, the AER-packet is converted to a spike-packet and broadcasted to all CNs of the system. Thus, a spike packet sent by a CN to the MN takes exactly the same route as a barrier-message sent by the same CN to MN. On the MN, AER-packets and barrier packets are received *in-order*. On the CN’s receiver side, both the received sync packet and the received spike packets are enqueued in the same input FIFO, cf. Figure 5A. So, the sync-message is detected by the control logic *after* the last spike packet has been fetched from the FIFO. Spikes which are valid for the next algorithmic time step are allowed to enter the FIFO before the control logic leaves the barrier and are processed as soon as the sync message has been removed from the FIFO.

#### CsNNs Microarchitecture and Logic Design

**FIGURE 7:**
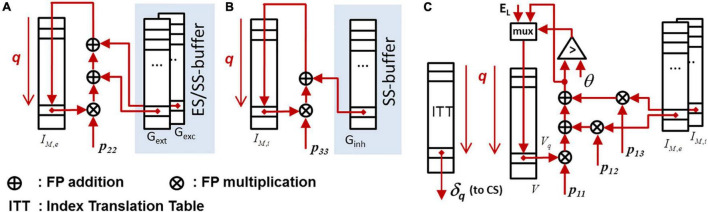
Data paths for exact ODE-integration. The underlying system matrix *P = {p_*ij*_}* representing the ODE-system (1) and (2) has a upper-triangular structure. The state memory is represented *by I_*M,e*_, I_*M,i*_*, and *V*, cf. (1) and (2). Detected spikes are first blocked in the output FIFO and are subsequently released when the router unit is able the process the next spike. **(A)** Data path for updating the lumped excitatory synaptic input. **(B)** Data path for updating the lumped inhibitory synaptic input. **(C)** Data path for updating the membrane voltage.

In the following, the logic design methodology will be briefly explained on the example of the ODE-solver unit (cf. section “The CsNNs Compute Node and Simulation Flow”). A straight-forward data flow implementation and memory organization is shown in Figures 7A–C. There is quite some architectural optimization potential by applying pipelining, parallelism and multiplexing in time in order to improve efficiency in terms of performance and hardware costs. However, the elaboration and evaluation of (many) design variants based on register-transfer-level (RTL) designs can be cumbersome and time consuming. This holds especially in light of the need of appropriate and non-obvious scheduling schemes, controlling and multiplexing of individual data flows, and including the overall design verification.

High-Level-Synthesis (HLS) appears to be an attractive design methodology allowing to conduct fast design experiments. Although, typically not the whole optimization potential can be exploited by HLS, it offers a trade-off between design time and efficiency, allowing for a quantitative design-space exploration. In HLS, the synthesis of behavioral descriptions are guided by so-called *synthesis directives*, specifying, e.g., memory port configurations, operand formats (i.e., single precision floating point) and data structures, latency constraints, and initiation intervals (*II*) in pipelined data paths (Xilinx, 2018a). The latter, combined with an appropriate choice of the memory interface configuration, directly impacts the degree of operator sharing. In addition to the actual data path, the complete control logic including standardized interfaces is provided by HLS. This significantly facilitates the integration and replacement of respective logic blocks in existing designs. Table 1 shows the results of a design space exploration experiment for various ODE-solver units and parameterizations based on a block processing 32 LIF neurons in a loop-type fashion (i.e., for 256 neurons per CN, eight parallel blocks have to be provided). It clearly shows available trade-offs between performance (in terms of latency and throughput) and logic effort (in terms of instantiated operators): Dependent on the given design goal (e.g., minimization of latency, minimization of resource utilization, etc.) different parameterizations emerge to be optimal. As an example, the minimization of *latency* is discussed in the following under the constraint of limited FPGA-resources.

**TABLE 1:** Results of a simple design space exploration of the ODE solver for different variants of synaptic shapes and parameterizations assuming a LIF neuron.

Synapse type (2 lumped synapses)	Matrix exponential and state vector	# Operators			*f*_clk_ = 150 MHz			
		FMUL	FADD	FCMP	II	IL	L_32_	T_32_[ms]
CUBA, exponential decay	$\left[\begin{array}{ccc} p_{11} & p_{21} & p_{31}\\ 0 & p_{22} & 0\\ 0 & 0 & p_{33} \end{array}\right]\cdot \left[\begin{array}{l} V_{q}\\ I_{e}\\ I_{i} \end{array}\right]$	1	1	1	5	40	197	1.31
		2	2	1	4	36	161	1.07
		2	2	1	3	39	133	0.89
		3	3	1	4	36	99	0.66
		5	5	1	1	36	69	0.46
CUBA, α/β-shape	$\left[\begin{array}{lllll} p_{11} & p_{21} & p_{31} & p_{41} & p_{51}\\ 0 & p_{22} & p_{32} & 0 & 0\\ 0 & 0 & p_{33} & 0 & 0\\ 0 & 0 & 0 & p_{44} & p_{54}\\ 0 & 0 & 0 & 0 & p_{55} \end{array}\right]\cdot \left[\begin{array}{l} V_{q}\\ I_{e}\\ Z_{e}\\ I_{i}\\ Z_{i} \end{array}\right]$	1	1	1	11	48	390	2.60
		2	1	1	10	44	356	2.37
		2	1	1	9	44	325	2.17
		2	2	1	8	44	294	1.96
		2	2	1	7	45	264	1.76
		2	2	1	6	46	234	1.56
		3	2	1	5	44	201	1.34
		3	3	1	4	44	169	1.13
		4	3	1	3	43	137	0.91
		6	5	1	2	43	106	0.71
		11	9	1	1	43	75	0.50

*A pipeline for operating 32 LIF neurons was specified for various synaptic kernels; II, initiation interval; IL, iteration latency; L_32_/T_32_, overall latency in clock cycles/μs to operate 32 neurons.*

Assume a block which is operated in a loop-type fashion and used to update the state of *N*_*p*_ neurons. The overall latency *L*_*Np*_ (in clock cycles), which is required to have all states updated after the initiation of the block, is given by *L_*Np*_ = IL + N_*p*_×II*. If *N* neurons are hosted in total on a CN and *B* blocks operate in parallel, *N*_*p*_ = ⌈*N/B*⌉ neurons can be assigned to each block. If a particular hardware resource *R*∈{LUT, FADD, FMUL, DSP, BRAM} on a FPGA is limited to *M*_*R*_ units and a block with initiation interval *II* requires *M*_*II,R*_ units for its implementation, the number *B* of implementable parallel blocks is *B* = ⌊*M_*R*_/M_*II,R*_*⌋. The optimization of latency then relies on the minimization of:


(5) $$\text{L}=\text{IL}+\text{II}\cdot \left\lceil N/\left\lfloor M_{R}/M_{II,R}\right\rfloor \right\rceil$$

The iteration latency *IL* is almost independent from the choice of the initiation interval *II* (exemplarily cf. Table 1). *IL* is related to the *critical path* of the underlying algorithm, while *II* is related to *operator sharing* inside the block. The choice of larger values for *II* allows for assigning multiple *algorithmic* operations to an *implemented* operator, which potentially saves logic resources. However, for *II* = 1 the number of implemented operators *M*_1,R_ and the number of algorithmic operations are necessarily on par. For larger values of *II* the inequality *M*_*II,R*_ ≥ ⌈*M_*II* = 1,R_/II*⌉ holds. Actually, the minimization of (5) requires the evaluation of architectural variants based on extensive variations of *II*. However, in many cases a good strategy is to make *II* as small as possible, while the number of parallel blocks *B* has to be as large as possible. In some cases, the choice *II* > 1 may break the blocks into smaller pieces (i.e., instantiated operators) which may better (i.e., closer) fit to the available amount of resources. This is especially useful if a single block with *II* = 1 cannot be implemented due to lack of resources. Based on the results of the extensive design space exploration, an optimal solution can be selected, which sufficiently fulfill performance demands and resource requirements. A detailed performance evaluation of various neuron models and ODE solvers is given in section “Performance for Alternative Neuron Models and Ordinary Differential Equation Solvers.”

#### CsNNs Network Generation

In order to evaluate the performance of CsNNs on INC-3000, the microcircuit was implemented. The parameters for the exact-exponential-integration ODE solver (initial states, factors, thresholds, and setup of the delay buffer), parameters for the on-node RTR unit (seeds for the TBRNGs, sequence lengths *L*_*i*_), and parameters for the router setup were determined off-line on the host PC. The host PC comprises an Intel Xeon Gold 6130 CPU running at 2.1 GHz and has 48 GB main memory clocked at 2,666 MHz. The starting point of configuration is a NEST description of the microcircuit (77,169 neurons, divided into eight populations, and 0.3 billion synapses). This network is first translated into a list of one-to-one connections between presynaptic neurons and postsynaptic neurons comprising individual values for the synaptic strength *J*, the synaptic delay *D*, presynaptic neuron index δ, and postsynaptic neuron index *N*. The set of neurons is partitioned in accordance to the population membership and uniformly distributed amongst a set of 305 CNs. Based on the particular assignment of neurons to nodes, specific sub-networks are extracted from the list-based network description. The individual statistics for the distribution of synaptic strength, the distribution of synaptic multiplicity, and the delay distribution were extracted from the sub-networks and algorithmically transformed into a parameterization for the MTBRNGs of the RTR units. Based on the MTBRNG parameterization appropriate sequence lengths *L*_*i,k*_ were algorithmically optimized for any possible presynaptic neuron δ_*p*_ and for any possible compute node *k*. Then, initial conditions were randomly set to the state memory of the ODE solver and the delay buffer of the RTR unit. Parameters for the TBRNGs representing the external input were derived based on a Poisson-distribution, population specific firing rates, and synaptic fan-in. Here, the TBRNGs are capable of representing 32 distinct output values which is equivalent to a maximum spike count of 31 input spikes per neuron and time step. For each node, the complete parameterization requires a memory amount of approximately 1.14 MB, while the whole network representation sums up to approximately 348 MB. The initial network representation would require more than 2.5 GB of memory, but the MTBRNG-representation of the network achieves an effective compression factor of 7. The TBRNG-based representation not only speeds up the on-node routing, it also speeds up the configuration of the INC-3000 system. While the generation of the configuration data on the host PC takes approximately 115 s wall-clock time, the upload of the configuration to INC-3000 sums up to less than 96 s, only.

## Results

### Breakdown of FPGA-Resources

**FIGURE 8:**
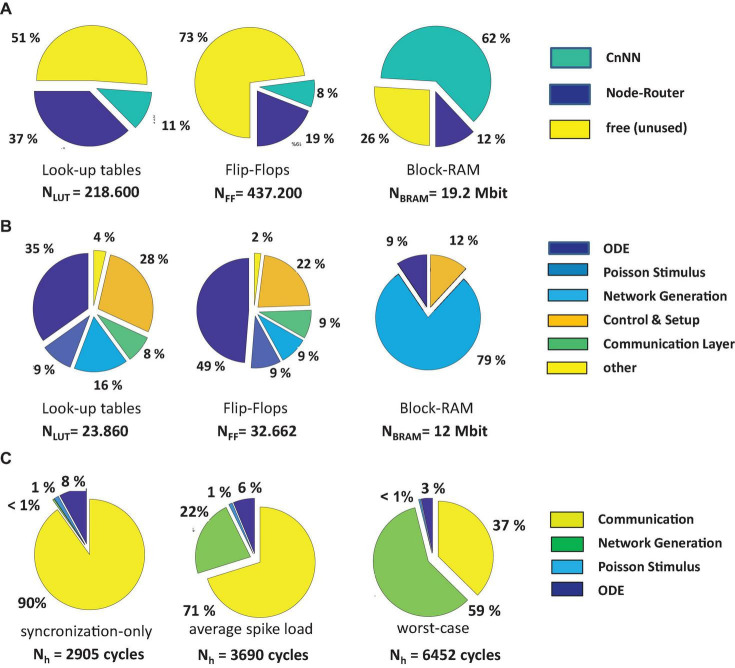
Overall resource breakdown including the node router logic **(A)**, resource breakdown of the CsNN architecture, without node router logic **(B)**, breakdown of the time step latency **(C)**.

Figures 8A,B shows the resource breakdown of the implemented circuit referring to Figure 3A. After synthesis and Place-and-Route 49% of the lookup-tables, 27% of the flip-flops, 74% of the BRAMs, and 12% of the DSPs were used for the realization of the CsNN architecture. While the CsNN architecture covers only a small fraction of the logic resources (LUTs and FFs), a large portion of the available block-RAM is allocated. Figure 8B reveals that most of the BRAM memory is integrated in the network generation unit (RTR) which in particular holds the seed tables representing the on-node network architecture. Even though the MTBRNG-based approach provides a significant reduction of required memory space for representing random networks the memory still remains (now resource-wisely) the critical element in the architecture.

The CsNN-architecture features eight parallel pipelines for implementing the ODE solvers and the Poisson spike generators (each serving up to 32 target neurons) and one pipeline for the RTR unit. The resources for each pipeline appear to be almost evenly distributed. Note that a very simple ODE-system has to be solved on one hand and the simple CB has been implemented in the RTR-unit. As it will be shown in section “Performance for Alternative Neuron Models and ODE Solvers,” the relative breakdown will change when complex neuro-synaptic models will be implemented.

### Performance

In order to validate the CsNNs network-implementation approach and to simultaneously determine its performance on INC-3000, the system was configured to simulate 15 min. of biological real time (9,000,000 time steps of Δ*t* = 100 μs). Each node was configured to store the locally generated spikes in the attached DDR3-memory (cf. Figure 3A). Several cycle counters were integrated and attached to the node logic which records the elapsed cycles between consecutive logical time steps. Figure 6D shows the result for the overall INC-3000 performance. The distribution of elapsed clock cycles shows a more or less narrow course. The number of synapses per axon is subject to variation (reflected by the *L*-parameters), which is also confirmed by Figure 6E showing the elapsed number of cycles to unroll the synaptic connection structure. On average, 3,695 clock cycles elapse between successive algorithmical time steps, or equivalently 24.63 μs (at 150 MHz clock frequency). Since an algorithmic time step represents Δ*t* = 100 μs the proposed hardware achieves a speed up factor of more than 4.06 which is – to the best of our knowledge – significantly larger than that of presently available digital hardware platforms (Knight and Nowotny, 2018; van Albada et al., 2018; Kurth et al., 2020; Rhodes et al., 2020).

Figure 8C shows the breakdowns of elapsed clock cycles between consecutive time steps under three different conditions. In Figure 8C (synchronization-only) a condition is shown in which none of the compute nodes has delivered a spike. Consequently, the RTR-unit is inactive in this time step. The time step latency is determined by the external stimulus generator, the latency of the ODE-unit, and the synchronization latency. Clearly, this condition represents the best case condition comprising the minimum time step latency. On the contrary, in Figure 8C (worst-case) a condition is shown which is characterized by a high spike occurrence. Then, on each node, the local RTR units create the node-local interconnect structure for each incoming spike event. Here, the cycle latency is determined by the particular node for which the elapsed cycles of the RTR unit accumulate to the maximum number. Compared to the average case (Figure 8C, average-spike-load) the observed worst case requires ×1.75 clock cycles to finish a simulation time step. This, however, illustrates the advantage of implementing a means for node synchronization. In a system with fixed wall clock time for stepping from one time step to the next a kind of worst case condition has to be predefined. While this particular worst case condition could be hard to determine in advance, a certain chance exists that this condition is underestimated which has the direct consequence of a potential spike loss. An overestimation, in turn, results in performance loss. On INC-3000, the proposed barrier-based node synchronization guarantees communication without spike loss and allows for a performance with average time step latency. This finally results in a speed-up factor of at least × 1.75 compared to an approach with a fixed wall clock.

#### Performance for Alternative Neuron Models and Ordinary Differential Equation Solvers

In the following, as an add-on to the performance evaluation of the standard LIF-model, the minimization of latency was elaborated for various linear and non-linear neuro-synaptic models and elaborated ODE-solver strategies.

While for purely linear models [LIF and MAT2 (Yamauchi et al., 2011), both with CUBA-based synaptic coupling], the method of exact integration (Rotter and Diesmann, 1999) can be applied, but non-linear ODEs require particular numeric strategies. Here, the method of Parker-Sochacki (PS) (Stewart and Bair, 2009) was implemented to solve a non-linear ODE system. PS-*q* evaluates the *exact* coefficients of the Maclaurin series of the sought solution up to a predefined order *q*. For the evaluation of PS-*q* the range of *q* was set to 1 ≤ *q* ≤ 6. A standard-Runge-Kutta integrator of order 4 (RK4) was implemented for comparison to PS-4 which shows up to perform slightly worse. The modified-Euler integrator (RK2) and the standard forward-Euler integrator (RK1) were finally compared against PS-2 and PS-1, respectively. Three types of non-linearities were considered: conductance-based synaptic coupling (COBA), the membrane dynamics of neurons defined by Izhikevich (2003), and the membrane dynamics of neurons defined by the adaptive exponential IAF model (Brette and Gerstner, 2005). For the synapses, δ-shape, exponential decay, and α/β-shaped kernels were used (two lumped synapses: excitatory and inhibitory) in order to evaluate ODE-systems with different number of coupled dynamic variables.

For the subsequent evaluation of models and solver strategies, *M*_*LUT*_ and *M*_*DSP*_ (cf. section “CsNNs Network Generation”) were specified which define the upper limit of permissible resources for the implementation of a given ODE-system and neuro-synaptic model, respectively. Within the chosen set of ODE-systems and applied solvers (cf. Supplementary Tables 1–3) the most resource-demanding neuro-synaptic model was given by a LIF-neuron with COBA-coupled α/β-shaped-synaptic kernels handled by PS-6. Here, a single implemented pipeline with *II* = 1 consumes 17% of the LUT-resources (*M*_*LUT*_ = 37,073 units) of the FPGA and 58.22% of the available DSP-units (*M*_*DSP*_ = 524 units) for the implementation of floating-point operators. Note that, by this particular choice, in any case enough FPGA resources were left for the additional implementation of the router logic and the communication system. Also note that the implemented ODE-units contain the complete logic for spike-detection, handling of refractory periods, and unit-configuration.

For a particular ODE-system *i*, the minimization of the latency was carried out such that both *M_*LUT*_* ≥ *M*_*LUT,i*_ and *M_*DSP*_ ≥ M_*DSP,i*_* was kept under variation of the initiation interval *II*. However, the choice *II* = 1 always lead to the shortest latency for all examined cases. For the more complex cases (i.e., non-linear ODE-systems and high-order solver) both *M*_*LUT*_ and *M*_*DSP*_ equally defined the resource limitations which indicates that the ratio *M*_*LUT,i*_/*M*_*DSP,i*_ approaches a constant here. On the contrary, simpler ODE-systems and/or low-order solvers require more LUT-resources in proportion to the DSP-resources which is mainly caused by the overhead for the attached logic used for spike-detection, setup, and control.

Based the experimental performance results (Figure 6) and synthesis results (Supplementary Tables 1–3, parameter *T*_256_) a particular speedup factor *G*_*BRT*_ is obtained for the various analyzed ODE units. The projection is valid on the assumption that the first-order spike statistics is invariant with respect to the underlying neuro-synaptic model (which requires a dedicated parameterization), the execution of the ODE-unit is in the critical path (which is given for the current implementation), and the ODE-unit is executed only once within the time-step Δ*t* ∼ 0.1 ms (i.e., there is no time-step subdivision). However, for some ODE-solver such as PS-1 sub-stepping to smaller values of Δ*t* may be necessary in order to keep numerical errors within tolerable bounds. Sub-stepping is excluded from the discussion. The results show that the performance of the INC-3000 system varies about 7% even under the assumption of relatively complex neuro-synaptic models: the latency for updating the complete set of state variables significantly falls below the synchronization latency in the communication system. Although the ODE-solver participates in the critical path of the overall simulation loop, its impact can be almost neglected compared to the impact of communication.

### Correctness

Neural network models and their simulations are critical targets for verification and validation, particularly when implemented on specialized hardware architectures. Implementation-sensitive details, such as the numerical precision of mathematical operations carried out on the hardware, the choice of algorithms (e.g., the choice of an ODE solver method) as well as simulation and model parameters have an effect on the correctness of the simulation outcome. Inappropriately implemented or chosen, they finally can induce deviations in network dynamics and change the characteristics of population-wide neural network activity. Even for domain experts it can become difficult to judge the correctness of the simulation results. This is all the more true in the absence of experimental validation data, such as biological data from electrophysiological recordings to define the ground truth.

In the following we describe the verification and validation approach used to demonstrate the correctness of the microcircuit implementation on the IBM INC-3000 Neural Supercomputer.

#### Methods

Both the correctness of the technical implementation, i.e., that each component does what it is supposed to do, and the consistency of the predictive simulation outcome with a defined ground truth are crucial aspects. The processes of ensuring this are referred to as verification^1^ and validation^2^. Together they accumulate evidence of a model’s correctness or accuracy (Thacker et al., 2004; Gutzen et al., 2018; Trensch et al., 2018).

In order to describe the characteristics of a neural network’s behavior, specific statistics are calculated to quantitatively capture features of the network activity. These measures also provide the tools for a systematic validation process. In the following we outline three measures of increasing complexity whose distributions were used for validating the simulation outcome of the microcircuit implementation.

*Average firing rate*: The average firing rate defines a measure to characterize the level of the network activity


(6) $$\text{FR}=\frac{n_{sp}}{T},$$

where *n*_*SP*_ is the number of spike events during time interval *T*.

*Coefficient of variation*: The measure analyzes the variability of the inter-spike intervals


(7) $$\begin{array}{c} CV=\frac{\sqrt{\frac{1}{n-1}\sum_{i=1}^{n}{\left(ISI_{i}-\bar{ISI}\right)}^{2}}}{\bar{ISI}}\;,\\ ISI_{i}=t_{i+1}-t_{i},\bar{ISI}=\frac{1}{n}\sum_{i=1}^{n}ISI_{i}, \end{array}$$

where *n* is the number of inter-spike intervals *ISI*_*i*_. In (7), *t*_*i*_ denote the ordered spike times of a neuron and $\bar{ISI}$ is the mean *ISI* (Shinomoto et al., 2003).

*Pearson’s correlation coefficient*: The pairwise Pearson’s correlation coefficient defines a measure that quantifies the temporal correlation of two binned spike trains (*i, j*) at the desired bin size. It is defined by the equation


(8) $$\text{C}\left[\text{i},\text{j}\right]=\frac{<{\text{b}}_{\text{i}}-\mu_{\text{i}},b_{\text{j}}-\mu_{\text{j}}>}{\sqrt{<{\text{b}}_{\text{i}}-\mu_{\text{i}},b_{\text{i}}-\mu_{\text{i}}>\cdot <{\text{b}}_{\text{i}}-\mu_{\text{i}},b_{\text{j}}-\mu_{\text{j}}>}},$$

where <.,.> is the scalar product of two vectors. *b*_*i*_ and *b*_*j*_ denote the binned spike trains, and μ_*i*_ and μ_*j*_ are their respective means (Gruen and Rotter, 2010).

In order to quantify the temporal correlation of all spike trains within a population the *N × N* matrix of the pairwise Pearson’s correlation coefficients between all combinations of *N* binned spike trains is calculated.

#### Verification

To achieve sufficient numerical accuracy on the CNs of the IBM INC-3000, the following design decisions have been made: (a) all calculations are performed in 32-bit single precision, (b) for the ODE solver method an exact integration scheme was implemented, and (c) the time resolution of the grid-based neural network simulation was set to 0.1 ms, which corresponds to the smallest connection delay in the microcircuit model. These design decisions are consistent with microcircuit model implementations using well-established neural network simulation tools such as NEST (Gewaltig and Diesmann, 2007) or the SpiNNaker neuromorphic system (Rhodes et al., 2020). Note, that SpiNNaker uses a 32-bit fixed-point format for representing state variables.

The high-level synthesis (HLS) logic design methodology allows the description of the microcircuit model in the C language, which is briefly described in section “CsNNs Microarchitecture and Logic Design.” HLS abstracts from the register-transfer level (RTL) design methodology, hiding complex and difficult to verify hardware implementation details. Quality and correctness of the HLS compiler output depends on this C implementation. For a source code verification (confirming that the functionality it implements works as intended) as well as a calculation verification (identify and remove errors in numerical simulations) of the implementation, simulation and testing on the C-level are therefore sufficient and were carried out accordingly.

#### Validation

Due to the lack of experimental data we defined as a reliable ground truth the results obtained from a simulation of the cortical microcircuit using NEST simulator published in van Albada et al. (2018). In order to judge the correctness of the microcircuit implementation on the IBM INC-3000 Neural Supercomputer and quantify the accuracy of the simulation outcome we compared the results obtained from the NEST simulation with the results obtained from the IBM INC-3000 implementation. This methodology where a model implementation is compared with a reliable reference implementation of the same model was termed substantiation^3^ in Gutzen et al. (2018); Trensch et al. (2018).

The cortical microcircuit model simulation produces a transient phenomenon exhibiting higher network activity in the first 1,000 ms of the simulated time. Therefore, for both simulations, the 15 min. of network activity data used for analysis was captured after this transient to ensure the networks being in a stable state.

#### Comparison

**FIGURE 9:**
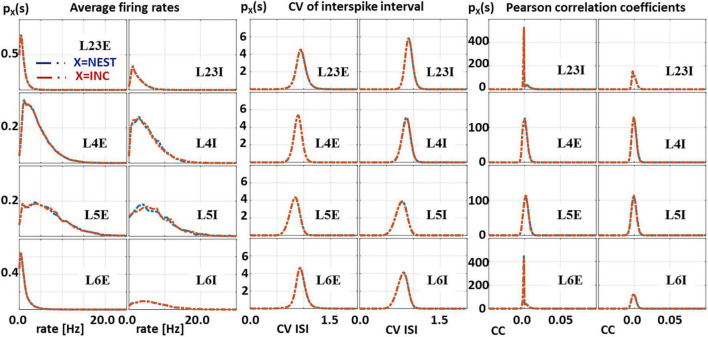
Substantiation assessment: Quantitative comparison of three characteristic measures from 15 min. network activity obtained from simulations of the cortical microcircuit on the IBM INC-3000 Neural Supercomputer and the NEST simulator. Probability distributions from left to right: average firing rate (FR), coefficient of variation (CV), and Pearson’s correlation coefficient (CC), spike trains are binned on a fine temporal scale of 2 ms. All measures for all layers of the cortical microcircuit are in close agreement.

We compared the distribution probabilities of three characteristic measures, the firing rates (FR), the coefficients of variation (CV), and the Pearson’s correlation coefficients (CC) calculated from 15 min. simulated time. The results are shown in Figure 9. For the calculation of Pearson’s correlation coefficient the spike trains were binned at 2 ms, i.e., much larger than the 100 μs quantization induced by the grid-based simulation. All measures for all populations of the cortical microcircuit are in close agreement and show statistical equivalence.

## Discussion

The INC-3000 neural supercomputer from IBM consists of 16 INC cards, each comprising 27 field-reconfigurable SoCs from Xilinx. Each SoC is connected to 1 GB of SDRAM and features both, a programmable logic system (PL, 218,600 lookup-tables, 437,200 flip-flops, 19.2 Mb memory) and an ARM A9-based processing system (PS, 2 ARM cores) as well. Additionally, each SoC comprises 16 serial transceivers with up to 12.5 Gbit/s data rate. Using these transceivers, the nodes of an INC-3000 machine are interconnected by a 12 × 12 × 3 mesh network topology. For the simulation of the cortical microcircuit only the programmable part (PL) of the SoC nodes was used. The arithmetic units (addition, multiplication, and comparison) are consistently implemented with single-float precision. A design implementation strategy based on high-level-synthesis (HLS) has been applied which allows for a fast design space exploration of various design parameterizations (e.g., the number of pipelines, degree of resource sharing, and memory interface configuration) and model/solver equations. First, the original cortical microcircuit comprising LIF neurons and CUBA-based exponential decay synapses was simulated with a speed-up of 4.06 with respect to biological real time using exact exponential integration. Here, a particular critical operation of the simulator architecture was given by the synapse-parameter look-up in the memory. Here, a novel network representation based on pseudo-random-number sequences was elaborated and implemented. Seeds for random number generators are used to represent small localized connectivity to target neurons, the associated individual weights, the synaptic delays, and synaptic multiplicity (also known as multapses). For each parameter (delay, weight, and multiplicity) almost arbitrary distribution functions can be preserved. In particular, continuous distribution functions were discretized using a variant of Walker’s method. The proposed method effectively results in a significantly reduced size of the data set required to setup the simulator by a factor of more than 7 while the setup time could be also significantly reduced to few minutes for a 306-node architecture. Note that the proposed method is not limited to static networks. In particular, in order to account for synaptic plasticity, the concept could be extended to be operated in a hybrid fashion – as already mentioned by Roth et al. (1997) – e.g., by storing synaptic weight values in the external RAM while the network connectivity and the dendritic delays are generated in parallel by procedural PRNG based operators, which could reduce the traffic to and from the memory system to the synaptic weights only. The validity of the proposed approach was successfully verified by a comparison with reference simulation results obtained from NEST. In order to verify the flexibility of the FPGA-based simulation architecture, networks with non-linear neuron models and conductance-based synaptic coupling were synthesized using the HLS-approach and evaluated with respect to performance and resource demands. Both, the Izhikevich neuron model and AdEx model in combination with exponential-decay, alpha-function shaped and beta-function shaped synaptic coupling were examined. In all cases, the simulation-time speed-up factor did not decrease by more than few percent. In particular, it turns out that latency constraints of the communication system of the IBM-INC machine limit the overall speed-up factor. The interconnect architecture of the INC-3000 system has been organized as a 3D-mesh comprising 12 × 12 × 3 nodes. The worst-case packet latency grows in proportion to the diameter of the underlying cuboid which includes the set of active compute nodes. While in highly structured networks the cruising range of a spike packet could be limited in the average case, the microcircuit effectively features almost-full-connection for all realizable ways of network decomposition. Therefore, any spike packet generated somewhere in the system has to be received by all other nodes as well. Fortunately, the spike packet latency depends on the spatial position of the packet-initiating node (nodes located in the center of the cuboid have shorter worst-case paths than nodes located at the edges). The situation appears to be different for the barrier-based node-synchronization. Here, each node has to submit a packet containing a barrier message to the MN (located in the center of the cuboid), and the MN broadcasts a SYNC-message back to all nodes. Therefore, the latency for the node synchronization appears to be equivalent to the worst-case latency given by the longest distance between nodes located in the corners of the cuboid. Nevertheless, it has been shown that the node-synchronization approach based on barrier messages has significant advantages. Node synchronization results in a further performance improvement by a factor of at least ×1.75 (average variable wall-clock timing compared to worst-case fixed wall-clock timing eventually including spike loss). Finally, the FPGA-based node architecture has great advantages for the implementation of advanced numerical strategies for solving complex ODE-systems. Restrictions mainly result from the limited amount of available BRAM-based local memory.

## Data Availability Statement

The original contributions presented in the study are included in the article/Supplementary Material, further inquiries can be directed to the corresponding author/s.

## Author Contributions

AH elaborated the concept of CsNN in cooperation with TN, implemented and verified CsNN on INC-3000, and main author of the article. TN elaborated the concept of CsNN in cooperation with AH. WW founded and leads the “Machine Intelligence” project at IBM Research, where the INC 3000 was conceived and build. CC was the main hardware designer of the IBM Neural Computer system. GP contributed by providing critical review, feedback, and proofreading of the manuscript. GT contributed to the validation and writing of the manuscript with section “Correctness.” MD contributed to the initial draft and edited the manuscript. All authors contributed to the article and approved the submitted version.

## Conflict of Interest

CC and WW were employed by IBM Research Division, Almaden Research Center, San Jose, CA, United States. The remaining authors declare that the research was conducted in the absence of any commercial or financial relationships that could be construed as a potential conflict of interest.

## Publisher’s Note

All claims expressed in this article are solely those of the authors and do not necessarily represent those of their affiliated organizations, or those of the publisher, the editors and the reviewers. Any product that may be evaluated in this article, or claim that may be made by its manufacturer, is not guaranteed or endorsed by the publisher.

## Footnotes

1 Verification describes the process of ensuring that the mathematical model is appropriately represented by the executable model, and improving this fit.

2 The validation process evaluates the consistency of the predictive simulation outcome with the system of interest. It aims at the agreement between experimental data that defines the *ground truth* and the simulation outcomes.

3 Substantiation describes the process of evaluating and quantifying the level of agreement of two executable models.
