# Supplementary material for: Simulating the Cortical Microcircuit Significantly Faster Than Real Time on the IBM INC-3000 Neural Supercomputer
Source: Front Neurosci. 2022 Jan 20;15:728460. doi: 10.3389/fnins.2021.728460 (PMC8811464; doi:10.3389/fnins.2021.728460)
Supplement: Supplementary file 1 [file Data_Sheet_1.pdf]

Supplementary Material

1 Supplementary Figures and Tables

1.1 Performance for Alternative Neuron Models and ODE Solvers

Performance data of the ODE-unit for integration of linear and non-linear ODE systems, after place-and-route on the FPGA are presented. Methods of *exact integration* (Rotter and Diesmann, 1999), *Parker-Sochacki integration* (Stewart and Bair, 2009), and *Runge-Kutta integration* were evaluated. The following acronyms were used: *IL*: iteration latency, *II*: initiation interval, *#P*: number of parallel pipelines, N_P: number of neurons assigned to a pipeline, #a_P/#m_P: number of floating-point-adders/multipliers per pipeline, #LUT/#DSP: number of allocated look-up-tables/DSPs after place and route, L_{256}/T_{256} : latency for updating 256 neurons in clock cycles/[µs] using #P pipelines, G_{BRT}: speedup-factor wrt. biological realtime.

Supplementary Table 1: Evaluation of the LIF model.

neuron	synapse	solver	IL	II	#P	N_p	#a_p	#m_p	#LUT	#DSP	L_{256}	T_{256}	G_{BRT}
LIF, CUBA	exp.decay	exact	36	1	11	24	5	5	34092	275	60	0,40	4,27
	α/β-shape	exact	43	1	8	32	9	11	32402	375	75	0,50	4,26
MAT-2, CUBA	exp.decay	exact	36	1	6	43	15	14	36519	432	79	0,53	4,25
	α/β-shape	exact	49	1	4	64	23	24	35852	472	113	0,75	4,21
LIF, COBA	α/β-shape	PS-6	234	1	1	256	85	81	29220	413	490	3,27	3,81
		PS-5	198	1	1	256	69	65	24880	333	454	3,03	3,84
		PS-4	148	1	1	256	54	50	19626	258	404	2,69	3,89
		PS-3	119	1	2	128	40	36	29578	376	247	1,65	4,06
		PS-2	76	1	4	64	27	23	36848	492	140	0,93	4,18
		PS-1	47	1	7	37	15	13	34390	441	84	0,56	4,24
		RK4	154	1	1	256	66	54	21833	294	410	2,71	4,05
		RK2	83	1	3	86	32	27	31628	435	169	1,13	4,14
		RK1	47	1	7	37	15	11	34303	441	84	0,56	4,24
LIF, COBA	exp. decay	PS-6	248	1	1	256	61	57	21926	293	504	3,36	3,79
		PS-5	212	1	2	128	49	45	32915	466	340	2,27	4,12
		PS-4	162	1	2	128	38	34	24805	356	290	1,93	4,01
		PS-3	133	1	3	86	28	24	26233	384	219	1,46	4,09
		PS-2	90	1	6	43	19	15	32418	498	97	0,64	4,23
		PS-1	61	1	11	24	11	7	29504	473	85	0,57	4,24
		RK4	168	1	2	128	44	34	29240	380	296	1,97	4,00
		PK2	97	1	5	52	22	17	36378	475	149	0,99	4,17
		PK1	54	1	11	24	11	7	38800	473	78	0,52	4,25

Supplementary Table 2: Evaluation of the Izhikevich model (Izhikevich, 2003).

neuron	synapse	solver	IL	II	#P	N_p	$\#a_p$	$\#m_p$	#LUT	#DSP	L_{256}	T_{256}	G_{BRT}
Izhikevich, CUBA	δ-shape	PS-6	206	1	2	128	48	42	30764	444	337	2,25	3,96
		PS-5	170	1	2	128	37	34	25746	360	301	2,01	3,99
		PS-4	134	1	3	86	30	26	30158	414	223	1,49	4,08
		PS-2	76	1	5	52	15	12	29716	345	131	1,07	4,15
		PS-1	54	1	8	32	10	6	33056	304	86	0,59	4,24
		RK4	140	1	3	86	35	28	34986	462	226	1,51	4,07
		RK2	82	1	6	43	17	14	32780	456	125	0,83	4,19
		RK1	49	1	11	24	10	6	33201	418	73	0,49	4,25
Izhikevich, COBA	exp. decay	PS-6	286	1	1	256	79	81	26878	410	542	3,61	3,75
		PS-5	236	1	1	256	62	63	21123	313	492	3,28	3,80
		PS-4	179	1	2	128	46	46	31270	460	307	2,05	3,99
		PS-2	93	1	5	52	21	17	33400	465	145	0,97	4,17
		PS-1	57	1	10	26	11	6	31718	400	83	0,56	4,24
		RK4	190	1	1	256	67	52	20397	290	446	2,97	3,85
		RK2	104	1	3	86	31	26	29642	420	190	1,27	4,12
		RK1	57	1	7	32	16	11	34003	455	89	0,59	4,24
Izhikevich, COBA	α/β-shape	PS-5	233	1	1	256	92	87	30466	445	489	3,26	3,81
		PS-4	169	1	1	256	71	66	23167	338	425	2,83	3,87
		PS-2	83	1	3	86	33	29	32149	459	169	1,13	4,14
		PS-1	47	1	6	43	17	13	32213	438	90	0,60	4,24
		RK4	176	1	2	256	91	72	27950	398	432	2,93	3,85
		RK2	90	1	3	128	40	36	26335	376	218	1,50	4,08
		RK1	43	1	5	52	19	15	30807	415	95	0,63	4,23

.

Supplementary Table 3: Evaluation of the adaptive-exponential IAF model (Brette and Gerstner, 2005).

neuron	synapse	solver	IL	II	#P	N_p	$\#a_p$	$\#m_p$	$\#e_p$	#LUT	#DSP	L_{256}	T_{256}	G_{BRT}
AdEx	δ-shape	PS-6	226	1	1	256	47	56	1	18641	288	490	3,26	3,81
		PS-5	193	1	2	128	38	44	1	30430	468	321	2,14	3,98
		PS-4	160	1	2	128	30	33	1	24204	370	288	1,92	4,01
		PS-2	108	1	5	52	17	14	1	34456	510	160	1,07	4,15
		PS-1	86	1	7	37	10	6	1	30757	448	123	0,82	4,19
		RK4	222	1	1	256	39	24	4	17475	298	480	3,20	3,81
		RK2	122	1	3	86	19	12	2	26346	444	208	1,39	4,10
		RK1	68	1	7	37	11	5	1	32360	518	105	0,70	4,22
AdEx, COBA	exp. decay	PS-6	285	1	1	256	98	101	1	35075	525	541	3,61	3,75
		PS-5	245	1	1	256	78	79	1	28104	419	501	3,34	3,79
		PS-4	198	1	1	256	60	59	1	21694	323	454	3,02	3,84
		PS-2	115	1	3	86	30	25	1	32849	483	201	0,75	4,21
		PS-1	86	1	5	52	17	11	1	32344	465	138	0,92	4,18
		RK4	261	1	1	256	71	56	4	28964	450	517	3,45	3,78
		RK2	131	1	2	128	33	28	2	28301	440	259	1,73	4,04
		RK1	62	1	4	64	17	12	1	27884	420	126	0,84	4,19
AdEx, COBA	α/β-shape	PS-4	198	1	1	256	76	75	1	27101	403	454	3,02	3,84
		PS-2	115	1	2	128	38	33	1	27695	402	243	1,62	4,06
		PS-1	86	1	4	64	21	15	1	31477	452	150	1,00	4,16
		RK4	261	1	1	256	95	76	4	36459	558	517	3,45	3,78
		RK2	131	1	2	128	43	38	2	35061	540	259	1,73	4,04
		RK1	62	1	4	64	21	16	1	33388	500	126	0,84	4,19

1.1.1 ODE solvers and HLS implementation

In the following, exemplary HLS-based implementations for particular ODE solver are shown. First, a brief overview of the underlying ODE solver methods is given. Both Parker-Sochacki (PS-4) and Runge-Kutta (RK-4) are considered.

1.1.1.1 The Parker-Sochacki-Method

An initial value problem (IVP) with autonomous ODE is stated as:

$$\dot{Y}(t) = F(Y(t)) \qquad Y(t_0) = Y_0 \tag{S1.1}$$

$$Y(t): R \rightarrow R^n \qquad Y_0 \in R^n \qquad F(Y): R^n \rightarrow R^n$$

The vector Y is called the *state vector*, the function F is called the *derivate*, Y_0 represents the initial values of Y at the initial time t_0, and t represents the (continuous) time.

Picard's method of successive approximation was designed to prove the existence of solutions to IVP:

$$Y^{(j+1)}(t) = Y_0 + \int_{t_0}^{t} F\big(Y^{(j)}(s)\big)ds \tag{S1.2}$$

In (S1.2) an iterated (approximated) solution $Y^{(j)}$ is used to find a better (improved) solution $Y^{(j+1)}$ for the considered time *t*. The goal of the Parker-Sochacki method (Parker, 1996) is to find a *Taylor-Series* representation of the time-dependent function, i.e. to determine coefficients Y_p

$$Y(t) = \sum_{p=0}^{\infty} Y_p \cdot t^p \tag{S1.3}$$

As soon as the coefficient are found, an update of *Y* for given time step *h* ($t=t_0+h$) is obtained by

$$Y(t_0 + h) = Y(t_0) + \sum_{p=1}^{n} Y_p \cdot h^p = \sum_{p=0}^{n} Y_p \cdot h^p \tag{S1.4}$$

As pointed out in (Stewart and Bair, 2009) it is always possible to *rescale* the ODE system such that the effective (numeric) step size equals 1 for a simulation with fixed physical time step *h*. Then (S1.4) reads as

$$Y(t_0 + h) = \sum_{p=0}^{n} Y_p \tag{S1.5}$$

1.1.1.2 The Runge-Kutta-Method

Runge-Kutta methods approximate the solution of (S1.1) by a weighted average of ***samples*** of the vector field *F(t,Y):*

$$Y_{j+1} = Y_j + h \cdot \sum_{i=1}^{m} \beta_i \cdot \widetilde{F}_i \tag{S1.6}$$

For an *explicit* Runge-Kutta method intermediate approximations of *F* are calculated by

$$\widetilde{F}_i = F\big(t_j + c_i \cdot h \ \ , \ Y_j + h \cdot \sum_{s=1}^{i-1} \alpha_{i,s} \cdot \widetilde{F}_s\big) \ \ , \ \ 0 \le c_i \le 1 \qquad . \tag{S1.7}$$

The method-dependent parameters are typically organized using a so-called Butcher tableau (Butcher, 2016), cf. **Supplementary Figure 1**.

The parameterization of RK-4 is

$$c_2 = c_3 = 0.5 \quad c_4 = 1.0 \quad \beta_1 = \beta_4 = \frac{1}{6} \quad \beta_2 = \beta_3 = \frac{1}{3} \quad \alpha_{2,1} = \alpha_{3,2} = 0.5 \quad \alpha_{4,3} = 1.0\ . \tag{S1.8}$$

All other entries in the Butcher-tableau are zero.

1.1.2 Izhikevich-Model with delta-shaped synaptic currents

The ODE system of the standard-Izhikevich model (Izhikevich, 2003), adapted from (Stewart and Bair, 2009), reads

$$C\dot{V} = k \cdot V \cdot (V - V_t) - U + I(const.) + I_{syn} \tag{S1.9}$$

$$\dot{U} = a \cdot (b \cdot V - U). \tag{S1.10}$$

In (S1.9, S1.10) *C, k,* V_t, *I(Const.), a*, and *b* represent constant model parameters. *V* represents the state of the membrane voltage, *U* represents the refractory state, and I_{syn} is the aggregated (excitatory and inhibitory) delta-shaped synaptic current. By rearranging terms and introduction of new constants (S1.9) and (S.10) can be rewritten as

$$\dot{V} = b_{12} \cdot V + b_{14} \cdot U + \quad b_{11} \cdot V^2 + b_{13} + \frac{I_{syn}}{C} \tag{S1.11}$$

$$\dot{U} = b_{21} \cdot V + b_{22} \cdot U \tag{S1.12}$$

using the definitions

$$b_{11} = \frac{k}{C} \quad b_{12} = -k \cdot \frac{V_t}{C} \quad b_{14} = -\frac{1}{C} \quad b_{13} = \frac{I}{C} \quad b_{21} = a \cdot b \quad b_{22} = -a\,. \tag{S1.13}$$

Parker-Sochacki. Based on the rules provided in (Stewart and Bair, 2009) the equation system (S1.11, S1.12) is translated into equations which provide the Taylor-coefficients for *U* and *V*, respectively:

$$V_1 = \left(b_{12} \cdot V_0 + b_{14} \cdot U_0 + \quad b_{11} \cdot (V^2)_0 + b_{13} + \frac{I}{C}\right) \cdot h \tag{S1.14}$$

$$V_{p+1} = \left(b_{12} \cdot V_p + b_{14} \cdot U_p + \quad b_{11} \cdot (V^2)_p\right) \cdot \frac{h}{p+1} \tag{S1.15}$$

$$U_{p+1} = \left(b_{21} \cdot V_p + b_{22} \cdot U_p\right) \cdot \frac{h}{p+1} \quad . \tag{S1.16}$$

In (S1.15) the term $(V^2)_p$ represents the so-called *Cauchy-product* which has to be applied for products of two dynamic variables *W* and *V* :

$$(W \cdot V)_p = \sum_{j=0}^{p} W_j \cdot V_{p-j} \quad . \tag{S1.18}$$

For given order *k*, the index *p* runs from *p*=1 to *p*=*k* which results in Taylor-coefficients $V_1 \ldots V_k$ and $U_1 \ldots U_k$. Both V_0 and U_0 are considered as initial conditions.

The iteration over *p* can be completely unrolled. For given time step *h* and index *p* all the coefficients

$$b_{ij\_p} \coloneqq b_{ij} \cdot \frac{h}{p+1} \quad p = 1..k \tag{S1.19}$$

are pre-computed in advance. In the given HLS-implementation, these coefficients are represented by the struct *mex_t* (cf. **Supplementary Figure 2A**). Then, the Taylor-coefficients (cf. **Supplementary Figure 2B**) are calculated by the unrolled iteration, which is depicted **Supplementary Figure 2C**.

Runge-Kutta. The evaluation of (S1.6) is based on four iterations and a final evaluation of a weighted sum (note: Y_0=*Y(t)*, $a_{m+1,m}$:= $a_{m,m-1}$):

$$k_1 = F(t_j, Y_0) \qquad \widetilde{Y_1} = Y_0 + \alpha_{2,1} \cdot h \cdot k_1 \tag{S1.20}$$

$$k_2 = F(t_j + c_2 \cdot h, \widetilde{Y_1}) \qquad \widetilde{Y_2} = Y_0 + \alpha_{3,2} \cdot h \cdot k_2 \tag{S1.21}$$

$$k_3 = F\left(t_j + c_3 \cdot h, \widetilde{Y_2}\right) \qquad \widetilde{Y_3} = Y_0 + \alpha_{4,3} \cdot h \cdot k_3 \tag{S1.22}$$

$$k_4 = F\left(t_j + c_4 \cdot h, \widetilde{Y_3}\right) \qquad \widetilde{Y_4} = Y_0 + \alpha_{5,4} \cdot h \cdot k_4 \tag{S1.23}$$

$$Y(t+h) = Y_0 + h \cdot (\beta_1 k_1 + \beta_2 k_2 + \beta_3 k_3 + \beta_4 k_4) \tag{S1.24}$$

Analogous to the case of Parker-Sochacki, equations (S1.20-S1.24) are considered as iterations, which unfold for the given ODE-system as:

$$V_{p+1} = V_0 + \left(b_{12} \cdot V_p + b_{14} \cdot U_p + \quad b_{11} \cdot V_p^2 + b_{13} + \frac{I}{C}\right) \cdot h \cdot \alpha_{p+2,p+1} \tag{S1.25}$$

$$U_{p+1} = U_0 + \left(b_{21} \cdot V_p + b_{22} \cdot U_p\right) \cdot h \cdot \alpha_{p+2,p+1} \tag{S1.26}$$

$$V(t+h) = \sum_{p=0}^{4} h \cdot \beta_i \cdot V_p \tag{S1.27}$$

$$U(t+h) = \sum_{p=0}^{4} h \cdot \beta_i \cdot U_p \tag{S1.28}$$

Note, that in (S1.27) and (S1.28) the definition $\beta_0 := 1/h$ was used in order to be consistent with (S1.24).

The iteration over *p* can be completely unrolled. For given time step *h* and index *p* all the coefficients

$$b_{ij\_p} \coloneqq b_{ij} \cdot h \cdot \alpha_{p+2,p+1} \quad p = 1..4$$

are pre-computed in advance. In the given HLS-implementation, these coefficients are represented by the struct *mex_t* (cf. **Supplementary Figure 3A**). Then, the intermediate approximations of *V* and *U* (cf. **Supplementary Figure 3B**) are calculated by the unrolled iteration, which is illustrated in **Supplementary Figure 3C**.

Structurally, RK and PS are similar. However, there are some fundamental differences. Binary products of state variables are replaced by Cauchy-products in PS. The update of the state vector requires a weighted sum in RK, while for PS only a summation of the considered Taylor-coefficients is needed in case the equation system was re-scaled to have an effective numerical step size of 1.

Embedding of the solver-methods. The methods described above are embedded in executable code that contains all important synthesis directives, memory structures, and variable definitions, cf. **Supplementary Figure 4**. All state variables are represented by single-precision-float variables (32 bit each) which are stored in a dedicated block ram. All state variables are combined in a single state vector (*mem.sum[]*). The core equations of the respective solver are embedded into a loop which is used to update the states of *prm.nSet* individual neurons. The loop body is pipelined and parameterized by II=1. External input (*mem.Gextern[]*), excitatory synaptic input (*mem.Gex[]*), and inhibitory synaptic input (*mem.Gin[]*) have to be updated before calling the function *ODE_SLV()*, e.g. by reading both the SS-buffer and ES-buffer (cf. **Supplementary Figure 3A**).

Before updating the state memory (*mem.sum[]*) spikes are detected (by comparing the final membrane potential V_k with the threshold *prm.vth*) and transmitted to the output FIFO (*mem.oFifo[]*). The fill level of the FIFO is represented by *mem.nOut*.

1.1.3 LIF neuron with conductance-based synaptic currents (COBA)

By integrating the conductance-based formalism into the IAF dynamics (Brette and Gerstner, 2007), the following equation system emerges for IAF-COBA with exponential decay:

$$C\dot{V} = -g_L \cdot V - \eta \cdot (V - E_\eta) - \gamma \cdot (V - E_\gamma) + I(const.) \quad \text{(S1.29)}$$

$$\dot{\eta} = -\lambda_\eta \cdot \eta \qquad \text{(excitatory lumped conductance)} \quad \text{(S1.30)}$$

$$\dot{\gamma} = -\lambda_\gamma \cdot \gamma \qquad \text{(inhibitory lumped conductance)} \quad \text{(S1.31)}$$

Both the lumped (excitatory) conductance η and (inhibitory) conductance γ are stepped by incoming spikes of respective type and decay exponentially. The parameters C, g_L, E_η, E_γ, V, λ_η, λ_γ represent the membrane capacitance, leakage conductance, excitatory reverse potential, inhibitor reverse potential, the membrane voltage, excitatory decay rate, and inhibitory decay rate of the model.

The re-arrangement of terms results in an equivalent representation of (S1.29-S1.31):

$$\dot{V} = l_{11} \cdot V + l_{12} \cdot \eta + l_{13} \cdot \gamma + n_{12} \cdot V \cdot (\eta + \gamma) + c_{11} \quad \text{(S1.32)}$$

$$\dot{\eta} = l_{22} \cdot \eta \quad \text{(S1.33)}$$

$$\dot{\gamma} = l_{44} \cdot \gamma \quad \text{(S1.34)}$$

using the definitions

$$l_{11} = -\frac{g_L}{C} \quad l_{12} = \frac{E_\eta}{C} \quad l_{13} = \frac{E_\gamma}{C} \quad n_{12} = -\frac{1}{C} \quad c_{11} = \frac{I(const.)}{C} \ . \quad \text{(S1.35)}$$

Parker-Sochacki. The solver is designed according to the rules described in section "Izhikevich-Model with delta-shaped synaptic currents". The equations for the Taylor-coefficients are given by:

$$V_1 = \left(l_{11} \cdot V_0 + l_{12} \cdot \eta_0 + l_{13} \cdot \gamma_0 + n_{12} \cdot \left(V \cdot (\eta + \gamma)\right)_0 + c_{11}\right) \cdot h \quad \text{(S1.36)}$$

$$V_{p+1} = \left(l_{11} \cdot V_p + l_{12} \cdot \eta_p + l_{13} \cdot \gamma_p + n_{12} \cdot (V \cdot (\eta + \gamma))_p\right) \cdot \frac{h}{p+1} \quad \text{(S1.37)}$$

$$\eta_{p+1} = \left(l_{22} \cdot \eta_p\right) \cdot \frac{h}{p+1} \quad \text{(S1.38)}$$

$$\gamma_{p+1} = \left(l_{44} \cdot \gamma_p\right) \cdot \frac{h}{p+1} \quad \text{(S1.39)}$$

The iteration over p can be completely unrolled. For given time step h and index p all the coefficients

$$l_{ij\_p} \coloneqq l_{ij} \cdot \frac{h}{p+1} \quad n_{ij_p} \coloneqq n_{ij} \cdot \frac{h}{p+1} \quad c_{ij\_p} \coloneqq c_{ij} \cdot \frac{h}{p+1} \quad p = 1..k \quad \text{(S1.40)}$$

are pre-computed in advance. In the given HLS-implementation, these coefficients are represented by the struct *mex_t* (cf. **Supplementary Figure 5A**). Then, the Taylor-coefficients (cf. **Supplementary Figure 5B**) are calculated by the unrolled iteration, which is depicted in **Supplementary Figure 5C**. Note that η is represented by *G1*, γ is represented by *G2*, and *V* is represented by *Vm*. *G1_0*, *G2_0*, and *Vm_0* represent initial values. *Vf1* represents the Cauchy-product between *V* and (η+γ).

Runge-Kutta. The solver is designed according to the rules described in section "Izhikevich-Model with delta-shaped synaptic currents". The emerging equations for RK-4 read:

$$V_{p+1} = \left(l_{11} \cdot V_p + l_{12} \cdot \eta_p + l_{13} \cdot \gamma_p + \ n_{12} \cdot V \cdot (\eta + \gamma) + c_{11}\right) \cdot h \cdot \alpha_{p+2,p+1} \quad \text{(S1.41)}$$

$$\eta_{p+1} = \left(l_{22} \cdot \eta_p\right) \cdot h \cdot \alpha_{p+2,p+1} \quad \text{(S1.42)}$$

$$\gamma_{p+1} = \left(l_{44} \cdot \gamma_p\right) \cdot h \cdot \alpha_{p+2,p+1} \quad \text{(S1.43)}$$

$$V(t+h) = \sum_{p=0}^{4} h \cdot \beta_i \cdot V_p \quad \text{(S1.44)}$$

$$\eta(t+h) = \sum_{p=0}^{4} h \cdot \beta_i \cdot \eta_p \quad \text{(S1.45)}$$

$$\gamma(t+h) = \sum_{p=0}^{4} h \cdot \beta_i \cdot \gamma_p \quad . \quad \text{(S1.46)}$$

The iteration over *p* can be completely unrolled. For given time step *h* and index *p* all the coefficients

$$l_{ij\_p} \coloneqq l_{ij} \cdot h \cdot \alpha_{p+2,p+1} \ \ n_{ij\_p} \coloneqq n_{ij} \cdot h \cdot \alpha_{p+2,p+1} \ \ c_{ij\_p} \coloneqq c_{ij} \cdot h \cdot \alpha_{p+2,p+1} \ \ p = 1..k \quad \text{(S1.47)}$$

are pre-computed in advance. In the given HLS-implementation, these coefficients are represented by the struct *mex_t* (cf. **Supplementary Figure 6A**). Then, the intermediate approximations of *V* and *U* (cf. **Supplementary Figure 6B**) are calculated by the unrolled iteration, which is illustrated in **Supplementary Figure 6C**.

Embedding of the solver-methods. The methods described above are embedded in executable code that contains all important synthesis directives, memory structures, and variable definitions, cf. **Supplementary Figure 7**. The design follows the principles described in section "Izhikevich-Model with delta-shaped synaptic currents".

1.2 Simple Example: Random Networks

Supplementary Figure 8A illustrates a simple network model proposed by Brunel (Brunel, 2000) which was intended to theoretically analyze the mutual interaction of an inhibitory population (*I*) with an excitatory population (*E*). Brunel has chosen simple linear models for membrane and synapse

dynamics aiming for an analytical treatment of describing the dynamical properties of randomly interconnected populations.

Populations can be understood as unordered sets of neurons sharing a common property e.g. with respect to particular synaptic parameters and connections to other populations. Here, neurons appear to be *unordered* in the sense that neurons are neither characterized by a spatial location nor a particular distance to other neurons. Populations are considered to be either excitatory (E) or inhibitory (I) which means that neurons that belong to a particular population develop synaptic connections to other neurons in the population-specific way exclusively. The size of a population X (i.e. the number of included neurons) is specified by the parameter N_X, $X \in \{E,I\}$. In random networks, the connections between a source population X and a target population Y is specified by the so-called connection density C_{YX}. If synaptic connections are drawn randomly and independently, the connection density and the number K_{YX} of synaptic connections are related by (Potjans and Diesmann, 2014)

$$\mathrm{C}_{YX} = 1 - \left(1 - \frac{1}{N_X \cdot N_Y}\right)^{K_{XY}}. \qquad \text{(S2.1)}$$

Note, that (S2.1) includes the chance of existence of multiple synaptic connections between a given pair of neurons (multapses). The potential existence of multapses in a network has significant impact on the design of algorithms and hardware structure in order to treat these appropriately. In addition to mutual connections between the populations so-called external stimuli are added to the network, mimicking the impact of adjacent networks which are not part of the actual network model. Typically, this impact is modeled as a directed process, i.e. the spike activity of the external stimuli impact the network activity but not conversely. Additionally, the external stimuli E_{ext} are typically modeled as being excitatory based, and featuring independent stationary (mostly) Poissonian spike trains with a constant average spike frequency ν_{ext}.

1.3 The Microcircuit Model

Supplementary Table 4: Parameters of the Microcircuit Model, notation after (Nordlie et al., 2009).

A	Model Summary
Structure	multi-layer excitatory-inhibitory (E-I) network
Populations	8 cortial populations in 4 layers (L2/3, L4, L5, L6)
Connectivity	random, independent, population-specific, fixed number of synapses
Neuron Model	leaky integrate-and-fire (LIF)
Synapse models	CUBA (exp.decay, alpha,beta) with static, normally distributed weights
Plasticity	none
Topology	none (no spatial information)
Delay Model	normally distributed delays
Input	independent stationary Poissonian spike trains
Measurements	spike times

B	Network Models
Connectivity	Connection probabilities C_{YX} from population X to population Y with<br>$\{X,Y\} \in \{L2/3,\ L4,\ L5,\ L6\} \times \{E,I\}$<br>fixed number of synapses S_{YX} between population X and Y<br>binomial distributed in-/out degrees<br>fixed in-degree for incoming external connections<br>autapses and multapses may emerge

C	Neuron Model
Cortex	**Leaky integrate-and-fire neuron (LIF)**<br>Dynamics of membrane potential $V_q(t)$ for neuron q<br>• Spike emission at times $t_{s,q}$ with $V_q(t_{s,q}) \geq V_\theta$<br>• Subthreshold dynamics<br>$C_m \cdot \frac{d}{dt} V_q(t) = -\frac{1}{R_m} \cdot V_q(t) + I_q(t) \quad if \ \ \forall s: t \notin \left]t_{s,q}, t_{s,q} + \tau_{ref}\right]$<br>• Reset and refractoriness:<br>$V_q(t) = V_{reset} \qquad if \ \ \forall s: t \in \left]t_{s,q}, t_{s,q} + \tau_{ref}\right]$<br>Random, uniform distribution of membrane potentials at $t = 0$
External input	Independent stationary Poisson point process with constant rate ν_{ext}

D		Connection Parameters and External Input
Symbol	Value	Description
J	87,81pA	Reference synaptic strength: all synapse weights are measured in units of J.
g_{XY}		Relative synaptic strength:
	1,0	$X \in$ {L2/3E, L4E, L5E, L6E}
	-4,0	$X \in$ {L2/3I, L4I, L5I, L6I}, except for:
	2,0	(X,Y)=(L4E, L2/3E)
$\sigma_{J,X,Y}$	$0{,}1 \cdot g_{XY} \cdot J$	Standard deviation of weight distribution
ν_{ext}	8,0 s^{-1}	Rate of external Poissonian input (cortico-cortical inputs)

E	Synapse Models
Weights	Distributed with static weights, clipped to preserve sign<br>$J_{qp} \sim N\{\mu = g_{XY} \cdot J \ \ , \sigma = \sigma_{J,X,Y}\}$
Delays	Distributed, left-clipped at 0<br>$D_{qp} \sim N\{\mu = \overline{D_X} \ \ , \sigma = \sigma_{d,X}\}$
Postsynaptic currents	Input current of neuron *q* from presynaptic neuron *p*<br>$I_{qp}(t) = J_{qp} \sum_{s} \eta(t - t_{s,p} - D_{qp}) \cdot \Theta(t - t_{s,p} - D_{qp})$<br>$t_{s,p}$ denotes the spike time of neuron *p*
Synaptic kernels	exponential decay $\eta(t) = \exp(-t/\tau_s)$<br>α-function $\eta(t) = \mathrm{t} \cdot \exp(-t/\tau_s)$<br>β-function $\eta(t) = [\exp(-t\ /\tau_{s,a}) - \exp(-t\ /\tau_{s,b})] \cdot \frac{\tau_{s,a} \cdot \tau_{s,b}}{\tau_{s,a} + \tau_{s,b}}$

F		LIF Neuron Model
Symbol	Value	Description
C_m	450 pF	Membrane capacitance
R_m	40 MΩ	Membrane resistance
E_L	-65 mV	Resistive leak reversal potential
V_θ	-50 mV	Spike detection threshold
V_{reset}	-65 mV	Spike reset potential
τ_{ref}	2,0 ms	Absolute refractory period after spikes
τ_s	0,5 ms	Postsynaptic current time constant

G	Connection Parameters	
Symbol	Value	Description
$\overline{D_E}$	1,5 ms	Mean excitator delay
$\overline{D_I}$	0,75 ms	Mean inhibitory delay
$\sigma_{d,X}$	0,5 $\overline{D_X}$	Standard deviation of delay distribution

H	Populations and external input								
Symbol	Value								Description
X	L2/3E	L2/3I	L4E	L4I	L5E	L5I	L6E	L6I	Name
N_X	20.683	5.834	21.915	5.479	4.850	1.065	14.395	3.948	Size
$K_{X,ext}$	1.600	1.500	2.100	1.900	2.000	1.900	2.900	2.100	External In-degree

I	Connection probabilites								
C_{XY}		from X							
		L2/3E	L2/3I	L4E	L4I	L5E	L5I	L6E	L6I
	L2/3E	0,1009	0,1689	0,0437	0,0818	0,0323	0,0	0,0076	0,0
	L2/3I	0,1346	0,1371	0,0316	0,0515	0,0755	0,0	0,0042	0,0
	L4E	0,0077	0,0059	0,0497	0,1350	0,0067	0,0003	0,0453	0,0
to Y	L4I	0,0691	0,0029	0,0794	0,1597	0,0033	0,0	0,1057	0,0
	L5E	0,1004	0,0622	0,0505	0,0057	0,0831	0,3726	0,0204	0,0
	L5I	0,0548	0,0269	0,0257	0,0022	0,0600	0,3158	0,0086	0,0
	L6E	0,0156	0,0066	0,0211	0,0166	0,0572	0,0197	0,0396	0,2252
	L6I	0,0364	0,0010	0,0034	0,0005	0,0277	0,0080	0,0658	0,1443

1.4 The CsNNs Spike-Distribution, Generation, and Synapse-Parameter Look-up

1.4.1 Locality Properties

At the scale of compute nodes the cortical microcircuit has a characteristic of a fully connected network. In order to see what this proposition means, let’s consider an (almost) arbitrary partitioning of the cortical microcircuit, i.e. the assignment of neuron to (compute) nodes in the INC-3000 system. Generally, the more compute nodes are considered (i.e. the less neurons are hosted by a particular node) the more the given network structure should have an effect on the choice of the optimal communication strategy. Here, the compute nodes were prepared with a capacity to host $|\underline{W}_k|\leq 256$ neurons per node while individual nodes host neurons with a definite population

membership. Note that the latter is the only restriction for the assignment of neurons to compute nodes for the moment. For a given population, neurons were uniformly distributed among the respective nodes. As a result, the full network can be represented by 305 compute nodes which means that more than 70% of the nodes in the INC-3000 system are participating in representing the cortical microcircuit.

If an arbitrary neuron is picked, it has connections to other neurons which are hosted by other compute nodes in the majority of cases. In order to quantify the connection density between compute nodes, the quantity P_{con} has been raised which quantifies the relative portion of compute nodes to which a given neuron has connections to (i.e. to postsynaptic neurons). **Supplementary Figure 8C** shows the result. On the x-axis, the normalized neuron index is mapped. '0.0' means 'neuron with index 0' while '1.0' means 'neuron with index 77168'. The diagram has been obtained by ordering with respect to P_{con}, i.e. the neuron comprising the highest value of P_{con} is located on the left hand side. By ordering, the diagram appears to be a continuous course and the indexing of neurons becomes arbitrary. $P_{con}=1$ means that connections exist on every node while $P_{con}=0$ indicates that there are no other connected nodes. The results show that more than 75% of the neurons have connections on *all* nodes, and only a small fraction of neurons show a structured (i.e. more directed) setup of connections. If the number of compute nodes is reduced, the characteristic converges more and more against an almost uniform distribution.

This observation is theoretically supported. For a network with an average connection probability p and a set of k postsynaptic neurons the probability of finding at least one connection for an arbitrarily selected presynaptic neuron can be approximated (under the assumption of a homogeneous distribution of connections) by

$$\mathrm{P}_1 \approx 1 - (1 - p)^k \qquad \text{(S3.1)}$$

P_1 vanishes in the case of small set size k, or in the case of a very sparse interconnect. However, for the exemplary value of p=5% (average connection density of the inner neurons in the microcircuit) and a threshold of P_1=50% (i.e. at least one connection exist in every second case) the set of postsynaptic neurons has to be smaller than $k < 14$ which is an unrealistically small number. All in all, the assumption of an almost fully connected network seems to be justified and obviously no advantage can be achieved for the communication scheme from any locality.

Independently from the applied partitioning, the cortical microcircuit shows a variation in the temporal density of spikes, cf. **Figure 8A** in the main document. Combining the variability of the spike count per time step and the variability of sequence length for the generation of the connection structure, a significant variability of the required physical processing time per time step can be expected for the system. Certainly, this variability is not only present from time step to time step but is also observable between various compute nodes which have different local interconnect patterns (e.g. due to different numbers of local synapses for given source neuron). The variability of physical processing time demands for a synchronization method which ensures that data and states which are distributed among the nodes via the communication system are processed at the right logical time step. In particular, spikes generated on a particular compute node k at node-local logical time step t_k should arrive at any other node q exactly when the local logical time step t_q coincides with t_k.

1.4.1.1 Workload balancing

In this work, each compute node hosts almost the same number of neurons which homogeneously splits the workload for updating the neuron dynamics. However, the main computation may be the synaptic integration operations, which may not be equally distributed for the nodes, since some neurons may have more synapses than others. In the following it is shown that the imbalanced distribution of synaptic connection results in a speedup penalty of less than 5%. The average number n_C of cycles used to handle the connection structure can be obtained from **Figure 8E** in the main document. Experimentally, we find n_C = 836 cycles in total for unpacking the local connection structure. Considering an iteration latency of IL=15 cycles and an initiation interval of II=1 cycle for the RTR unit, the cycle overhead due to an imbalanced distribution of synapses can be estimated. From **Figure 8A** in the main document an average amount of μ=24.6 spikes per time step is obtained which results in an average of

$$n_s = \frac{\left(\frac{n_C}{\mu} - IL\right)}{II} \approx 19$$

activated synapses per CN which are in the critical path. This is equivalent to a connection density of

$$c_k = \frac{n_S}{|W_k|} \approx 7.4\%.$$

The real average connection density is about $\underline{c_k} \approx$ 5% (0.3 billion synapses, 77169 neurons) which results in an estimated cycle overhead of 50% caused by the imbalanced distribution of synapses. Hence, in the optimal case $\underline{n_S} \approx \underline{c_k} \times |W_k|$ = 12.8 synapses per CN (equivalent to $\underline{n_c} = \mu \times (\underline{n_S} \times II + IL)$ $\approx$ 683 cycles on average or a saving of 153 cycles) would constitute a reasonable lower limit if only the connection densities are considered. Considering an average number of n_{cl} = 3695 elapsed clock cycles between logical time steps (cf. **Figure 8D** in the main document), an improvement of less than 5% for the overall acceleration factor would by possible. Consequently, due to the large communication latencies slightly imbalanced loads have only minor effects on the performance of CsNNs on INC.

1.4.2 Twin numbers, autapses, and multapses

Special attention has to be paid on the fact, that all derived numbers relevant for the network generation are practically drawn from a discrete distribution. As a consequence, the probability of finding *twin numbers* in a sufficiently long sequence is non-zero. As will be shown below, these *twin numbers* have to be detected and to be dedicatedly treated in particular cases.

The effect of observing twin numbers in discrete random sequences is strongly related to so-called *multapses* which denote multiple synaptic connections from a particular source neuron to a target neuron. Multapses naturally occur if target IDs of postsynaptic neurons are independently drawn from a distribution. **Supplementary Figure 9** illustrates the term *multapse*. Three source neurons *k*, *l*, and *r* are shown which are connected to three target neurons *v*, *u*, and *r* of a micro cluster. In the case that a neuron connects to itself (in the example: the connection *r*-*r*), the synaptic connection is called an *autapse*. In most cases (i.e. in networks with relatively low connection probabilities) the established synaptic connections are onefold, i.e. there is only one synapse between a pair of neurons. Nevertheless, if synaptic connections are drawn *independently*, a certain chance exists that pairs of neurons are connected by *multiple* synapses. In the given example, the connection q-v is twofold and

the connection q-u is threefold. Both connections establish a so-called *multapse*. Typically, *multapes* can be explicitly part of the network model. Generally, if good model description principles are applied, it needs to be specified if these type of connection is permitted or not (Nordlie et al., 2009). In the following, it is assumed that the synaptic multiplicity M generally follows a separate and independent *pmf* which is represented by an own MTBRNG.

An incoming spike δ_p is directly used to address so-called seed-tables (cf. **Figure 3B** in the main document). Individual seeds are selected and used for the initialization of the MTBRNGs located in the RTR-logic. Two additional parameters are read out which specify the overall length of the sequence (parameter L) and a specifier S which is used to select a particular set of (H,A,N)-tables of the MTBRNGs. The parameter S represents the population membership of the spike δ_p. By addressing a particular set of (H,A,N)-tables, networks with population-specific statistical properties can be maintained. The parameter L is (roughly) equivalent to the number of synapses on a given (local) axon. MTBRNGs are provided for the generation of a synaptic strength J, an (integer) synaptic transmission delay D, and a synaptic multiplicity M. Further, a PRNG is provided for the generation of an index N to a postsynaptic neuron. Note that each TBRND is capable of representing 64 distinct output values (of type 6-bit integer for a synaptic delay, and of type single precision float for a synaptic weight, and of type 8-bit integer for an index to a postsynaptic neuron).

In **Supplementary Figure 10** the unwinding of the local synaptic structure is shown for three cases (all related to **Supplementary Figure 9**). In the first case (source ID = p) L=3 iterations are executed to obtain the set of postsynaptic neurons N_k including synaptic delays D_k and weights J_k. In each cycle the generated values are transmitted to the circular buffer (CB) which updates the memory representing the synaptic input. In the second case (source ID = l), the PRNG (incidentally) generates a *twin number* for the postsynaptic neuron N in the second cycle (index to neuron u). Twin numbers for indexing postsynaptic neurons will superimpose the statistics for the synaptic multiplicity M and need to be avoided in order to prevent skewing the intended pmf of M. For that purpose, the RTR-logic keeps a list of postsynaptic neurons addressed so far and cancels an update of the CB in case of a match (mark $^{1)}$).

Note that the involved comparison can be organized to operate in a fully parallel way and causes no significant additional latency in the critical path. In the third case (source ID = r), the MTBRNG creates a twin number for the synaptic delay of a multapse with M=3. Generally, all synapses of a multapse are connected to the same postsynaptic neuron (here: N=u). Since the CB is addressed by N and the synaptic delay D as well, a twin number for the delay D would result in addressing a particular memory location in the CB *twice*. This poses a data-dependency issue, if the write operation to the CB of the first update has not finished before the initial read of the second update has been initiated. This, in particular, is given if the addition takes several cycles to finish an operation. Therefore, twin numbers for synaptic delays D found during unwinding a multapse are to be avoided (mark $^{2)}$) which is realized by keeping also a list of synaptic delays generated so far for the current multapse. All in all, the RTR-logic contains 3 MTBRNGs, a single PRNG, a finite state machine for controlling the iteration loop and two lists for detecting twin-numbers.

The individual settings for the (H,A,N)-tables of the MTBRNGs were found using a strategy described below. If the connection density C_{ij} (S.1) is non-zero, an initial sample connection structure between the populations i and j can be obtained using the standard simulation language interpreter (SLI) of NEST (Gewaltig and Diesmann, 2007) or PyNN (Davidson et al., 2008). Using NEST, connections are chosen in a random way applying a few restrictions and parameters. For given i and j

the connection structure can be described by three independent distributions: a pmf $P_M(m)$ for the synaptic multiplicity M, a pmf $P_D(d)$ for the synaptic transmission delays D, and a pdf $\underline{P}_J(k)$ for synaptic strengths J. The indexing (i,j) with respect to source and target population, respectively, was omitted here but it has to be kept in mind that all these functions effectively depend on i and j. Here, by the introduction of am pmf $P_J(k)$, it is possible - within certain bounds - to replace continuous synaptic strengths by a discrete approximation of the form

$$\mathrm{J} = J_0 + k \cdot \Delta J \quad , k \in \{0,1,2,\dots\} \quad P_J(k) = P(J = J_0 + k \cdot \Delta J) \qquad (S4.1)$$

In the subsequent discussion the continuous pdf $\underline{P}_J(k)$ is approximated by a discrete pmf $P_J(k)$.

In principle, the knowledge about the parameterization of all relevant pmfs should be sufficient to configure all MTBRNGs in the system. There are two ways to achieve the configuration: (i) To re-implement the generator functions on which the pmfs are based on, or (ii) to raise empirical histograms obtained by counting the frequency of occurrence of a particular feature (i.e. delay D, synaptic multiplicity M, and synaptic strength J) within an *a priori* existing network. In order to compare the performance of a standard-NEST implementation (i.e. the reference implementation) of the microcircuit and the proposed approach of network representation, the second concept was selected here in order to bring both *instantiated* networks in accordance as close as possible. Let $W_j = \{q_1, q_2, ..., q_{n_j}\}$ represent the set of neurons which belong to a population j. If the size of a given population is larger than the maximum number of neurons whose dynamics can be handled by a single node, the set $\underline{W}_k \subset W_j$ represents a true subset of W_j implemented on CN k, which establishes a micro-cluster, cf. sect. 2.3.1. In that way, given a micro-cluster mapped on node k, node-specific histograms of interesting features can be raised by counting. Based on the results, the *(H,A,N)*-tables of the node-local MTBRNGs can be directly configured from that data. The choice of the content of the seed tables is arbitrary but should be random. For any $q \in W_i$ the population membership is given by j which defines the S-table in the seed-table complex.

1.5 Supplementary Figures

$$
\begin{array}{c|ccccc}
0 & & & & & 0 \\
c_2 & \alpha_{2,1} & & & & 0 \\
c_3 & \alpha_{3,1} & \alpha_{3,2} & & & 0 \\
\cdots & \cdots & \cdots & & & \cdots \\
c_m & \alpha_{m,1} & \alpha_{m,2} & \cdots & \alpha_{m,m-1} & 0 \\
\hline
 & \beta_1 & & & \cdots & \beta_m
\end{array}
$$

Supplementary Figure 1: Organization of the Butcher tableau

A.

```
typedef struct

mex_tt {

float   b11_1;
float   b12_1;
float   b13_1;
float   b14_1;
float   b21_1;
float   b22_1;

float   b11_2;
float   b12_2;
float   b14_2;
float   b21_2;
float   b22_2;

float   b11_3;
float   b12_3;
float   b14_3;
float   b21_3;
float   b22_3;

float   b11_4;
float   b12_4;
float   b14_4;
float   b21_4;
float   b22_4;

} mex_t;
```

B.

```
/* begin variables definition */

float      Gxt,Gex,Gin,m2,m1;
uint32_t   aer;

union    {uint32_t i; float f;}  Vm,Um;
union    {uint32_t i; float f;}  Vm_nxt, Um_nxt;
uint64   ste, ste0,ste1;

float     Vk,Uk,V0,U0;
float     V1,U1,V0f;
float     V2,U2,V1f;
float     V3,U3,V2f;
float     V4,U4,V3f;

/* end variables definition */
```

C.

```
/* begin solver equations PS4 */

m1   = Gxt + Gex - Gin;
V0 = Vm.f + m1;
U0 = Um.f;

// coefficient of first iteration

V0f = V0 * V0;
V1 = (cnt->mex.b12_1 * V0 + cnt->mex.b14_1 * U0  +
        cnt->mex.b11_1 * V0f + cnt->mex.b13_1);
U1 = (cnt->mex.b21_1 * V0 + cnt->mex.b22_1 * U0);
```

C. (cont.)

```
// coefficient of second iteration

V1f = (V0 * V1) + (V1 * V0); // Cauchy-product
V2 = (cnt->mex.b12_2 * V1 + cnt->mex.b14_2 * U1 + cnt->mex.b11_2 * (V1f));
U2 = (cnt->mex.b21_2 * V1 + cnt->mex.b22_2 * U1);

// coefficient of third iteration

V2f = ((V0 * V2) + (V1 * V1)) + (V2 * V0); // Cauchy-product
V3 = (cnt->mex.b12_3 * V2 + cnt->mex.b14_3 * U2 + cnt->mex.b11_3 * V2f);
U3 = (cnt->mex.b21_3 * V2 + cnt->mex.b22_3 * U2) ;

// coefficient of fourth iteration

V3f = ((V0 * V3) + (V1 * V2)) + ((V2 * V1) + (V3 * V0)); // Cauchy-product
V4 = (cnt->mex.b12_4 * V3 + cnt->mex.b14_4 * U3 + cnt->mex.b11_4 * V3f);
U4 = (cnt->mex.b21_4 * V3 + cnt->mex.b22_4 * U3) ;

// sum-up
Vk = (V0 + V1) + (V2 + V3) + V4 ;
Uk = (U0 + U1) + (U2 + U3) + U4 ;


/* end solver equations PS4 */
```

Supplementary Figure 2: Core of the Parker-Sochacki HLS implementation. **(A)** pre-calculated constants, related to the underlying ODE system and solver. **(B)** declaration of the dynamic variables and coefficients of the Taylor-series, **(C)** unrolled PS-iterations of order 4.

A.

```
typedef struct

mex_tt {

float   b11_1;
float   b12_1;
float   b13_1;
float   b14_1;
float   b21_1;
float   b22_1;

float   b11_2;
float   b12_2;
float   b13_2;
float   b14_2;
float   b21_2;
float   b22_2;

float   b11_4;
float   b12_4;
float   b13_4;
float   b14_4;
float   b21_4;
float   b22_4;

float   c0;
float   c1;

} mex_t;
```

B.

```
/* begin variables definition */

float      Gxt,Gex,Gin,m1,m2;
uint32_t   aer;

union     {uint32_t i; float f;}  Vm, Um;
union     {uint32_t i; float f;}  Vm_nxt, Um_nxt;
uint64    ste,ste0,ste1;

float     Vk,Uk,V0,U0, V01, V00;
float     V1,U1,V0f,V10,V11;
float     V2,U2,V1f,V20,V21;
float     V3,U3,V2f,V30,V31;
float     V4,U4,V3f;
/* end variables definition */
```

C.

```
/* begin solver equations RK4 */

m1   = Gxt + Gex - Gin;
V0 = Vm.f + m1;
U0 = Um.f;

// first iteration

V0f =  V0 * V0;
V00= cnt->mex.b12_2 * V0 + cnt->mex.b14_2 * U0 ;
V01= V0 + cnt->mex.b13_2;
V1 = (cnt->mex.b11_2 * V0f + V01) + V00;
U1 = U0 +  (cnt->mex.b21_2 * V0 + cnt->mex.b22_2 * U0);
```

C. (cont.)

```
// second iteration

V1f = V1 * V1;
V10= cnt->mex.b12_2 * V1 + cnt->mex.b14_2 * U1 ;
V11 = V01;
V2 = (cnt->mex.b11_2 * V1f + V11) + V10 ;
U2 = U0 +  (cnt->mex.b21_2 * V1 + cnt->mex.b22_2 * U1);

// third iteration

V2f = V2 * V2;
V20= cnt->mex.b12_1 * V2 + cnt->mex.b14_1 * U2 ;
V21 = V0 + cnt->mex.b13_1;
V3 = (cnt->mex.b11_1 * V2f + V21) + V20 ;
U3 = U0 +  (cnt->mex.b21_1 * V2 + cnt->mex.b22_1 * U2);

// fourth iteration

V3f = V3 * V3;
V30=  cnt->mex.b12_4 * V3 + cnt->mex.b14_4 * U3 ;
V31 = V0 + cnt->mex.b13_4;
V4 = (cnt->mex.b11_4 * V3f + V31) +  V30 ;
U4 = (U0 +  cnt->mex.b21_4 * V3) + cnt->mex.b22_4 * U3;

// sum up

Vk = (cnt->mex.c1*(V2 + V4) + cnt->mex.c0 *(V1 + V3)) - V0;
Uk = (cnt->mex.c1*(U2 + U4) + cnt->mex.c0 *(U1 + U3)) - U0;

/* end solver equations RK4 */
```

Supplementary Figure 3: Core of the Runge-Kutta HLS implementation. **(A)** pre-calculated constants, related to the underlying ODE system and solver. **(B)** declaration of the dynamic variables and coefficients of the Taylor-series, **(C)** unrolled RK-iterations.

A.

```
#include <stdlib.h>
#include <stdio.h>
#include <stdint.h>
#include <math.h>
#include <ap_cint.h>

#define NSIZEPERPIPE 256
```

B.

```
#pragma HLS RESOURCE variable=cnt->mem.Sum core=RAM_T2P_BRAM
#pragma HLS RESOURCE variable=cnt->mem.Gextern core=RAM_1P_BRAM
#pragma HLS RESOURCE variable=cnt->mem.Gin     core=RAM_1P_BRAM
#pragma HLS RESOURCE variable=cnt->mem.Gex     core=RAM_1P_BRAM
#pragma HLS RESOURCE variable=cnt->mem.oFifo   core=RAM_1P_BRAM
```

C.

```
typedef struct prm_tt {

  float     Erest;     /* membrane V after spike          */
  float     vth;       /* membrane threshold              */
  float     dth;       /* parameter d in the IZH model */
  uint16_t  nSet;      /* number of active neurons      */
} prm_t;
```

D.

```
typedef struct mem_tt {
  float     Gextern[NSIZEPERPIPE];  /* from Poisson generator          */
  float     Gex[NSIZEPERPIPE];      /* excitatory lumped synapses   */
  float     Gin[NSIZEPERPIPE];      /* inhibitory lumped synapses    */
  uint64    Sum[NSIZEPERPIPE];      /* state vector                           */
  uint32_t  AER[NSIZEPERPIPE];      /* AER index table                     */
  uint32_t  oFifo[NSIZEPERPIPE];    /* output buffer for spikes          */
  uint16_t  nOut;                   /* pointer to empty line in oFifo   */
} mem_t;
```

E.

```
typedef struct cntnr_tt {
   mem_t     mem; /* memory structures   */
   prm_t     prm;  /* parameter              */
   mex_t     mex;  /* method-coefficients */
} cntnr_t;
```

F.

```
void ODE_SLV(cntnr_t *cnt) {

   uint16_t   neuronIndex, nOut;

   /* variables definition here */

  for (neuronIndex = 0 ; neuronIndex < cnt->prm.nSet ; neuronIndex++) {
#pragma HLS LOOP_TRIPCOUNT min=256 max=256
#pragma AP DEPENDENCE variable=cnt->mem.Sum inter false
#pragma HLS PIPELINE II=1

     nOut = cnt->mem.nOut;
     Gxt  = cnt->mem.Gextern[neuronIndex];
     Gex  = cnt->mem.Gex[neuronIndex];
     Gin  = cnt->mem.Gin[neuronIndex];
     aer  = cnt->mem.AER[neuronIndex];

     ste    = cnt->mem.Sum[neuronIndex];   /* get full state vector           */
     ste0  = apint_get_range(ste,  31,   0);   /* extract membrane voltage */
     ste1  = apint_get_range(ste,  63,  32);  /* extract refractory variable */
     Vm.i   = (uint32_t) ste0;
     Um.i   = (uint32_t) ste1;

      /* solver equations here */

      /* comparison with threshold, update state                */
      m2 = Uk + cnt->prm.dth;
      Vm_nxt.f           = (( Vk >= cnt->prm.vth) ) ? cnt->prm.Erest    : Vk;
      Um_nxt.f           = (( Vk >= cnt->prm.vth) ) ? m2                       : Uk;
      cnt->mem.nOut       = (( Vk >= cnt->prm.vth) ) ? nOut + 1        : nOut;

      // collect all the bits and form an 96 bit update

      ste0 = (uint64) Vm_nxt.i;
      ste  = apint_set_range(ste0,  63, 32, (uint64) Um_nxt.i);
      cnt->mem.Sum[neuronIndex]  = ste;
      cnt->mem.oFifo[nOut] = aer;

    } /* end for (neuronIndex = 0  */
} /* end ODE_SLV */
```

Supplementary Figure 4: (A) include libraries and defines. **(B)** synthesis directives for memories: true-2-port and single-port block RAMs. **(C)** structure for representing neuron parameters. **(D)** structure including memories which store state variables and synaptic input. **(E)** structure comprising memories, neuron parameters, and pre-calculated method-specific coefficients. **(F)** top-level solver.

A.

```
typedef struct

mex_tt {

float   l11_1;
float   l12_1;
float   l13_1;
float   n12_1;
float   c11_1;
float   l22_1;
float   l44_1;

float   l11_2;
float   l12_2;
float   l13_2;
float   n12_2;
float   l22_2;
float   l44_2;

float   l11_3;
float   l12_3;
float   l13_3;
float   n12_3;
float   l22_3;
float   l44_3;

float   l11_4;
float   l12_4;
float   l13_4;
float   n12_4;
float   l22_4;
float   l44_4;

} mex_t;
```

B.

```
/* begin variables definition */

float     Gxt,Gex,Gin,m1,m2;
uint32_t  aer;
union     {uint32_t i; float f;}  Vm, I1, I2;
union     {uint32_t i; float f;}  Vm_nxt,I1_nxt,I2_nxt;
uint96    ste,ste0,ste1,ste2;

// method variables
float  Vm_0, G1_0, G2_0;
float  Vm_1, G1_1, G2_1,  Vf1_0, Gs_0;
float  Vm_2, G1_2, G2_2, Vf1_1, Gs_1;
float  Vm_3, G1_3, G2_3,  Vf1_2, Gs_2;
float  Vm_4, G1_4, G2_4,  Vf1_3, Gs_3;
float  Vm_k, G1_k, G2_k;
/* end variables definition */
```

C.

```
/* begin solver equations PS4 */

m1   = Gxt + Gex + I1.f;
m2   = Gin         + I2.f;
Vm_0 = Vm.f;
G1_0 = m1;
G2_0 = m2;

// coefficient of first iteration

Gs_0  = G1_0 + G2_0;
Vf1_0 = Gs_0 * Vm_0;
Vm_1 =  (cnt->mex.l11_1 * Vm_0 + cnt->mex.l12_1 * G1_0) +
           (cnt->mex.l13_1 * G2_0 + cnt->mex.n12_1 * Vf1_0) +
           (cnt->mex.c11_1);
G1_1 =  cnt->mex.l22_1 * G1_0;
G2_1 =  cnt->mex.l44_1 * G2_0 ;
```

C. (cont.)

```
// coefficient of second iteration

Gs_1   = G1_1 + G2_1;
Vf1_1  = Gs_0 * Vm_1  +  Gs_1 * Vm_0; // Cauchy-product
Vm_2 =  (cnt->mex.l11_2 * Vm_1 + cnt->mex.l12_2 * G1_1) +
          (cnt->mex.l13_2 * G2_1 + cnt->mex.n12_2 * Vf1_1);
G1_2 =  cnt->mex.l22_2 * G1_1 ;
G2_2 =  cnt->mex.l44_2 * G2_1 ;

// coefficient of third iteration

Gs_2   =  G1_2 + G2_2;
Vf1_2  = (Gs_0 * Vm_2  +  Gs_1 * Vm_1)  +  Gs_2 * Vm_0; // CP
Vm_3 =  (cnt->mex.l11_3 * Vm_2 + cnt->mex.l12_3 * G1_2) +
           (cnt->mex.l13_3 * G2_2 + cnt->mex.n12_3 * Vf1_2);
G1_3 =  cnt->mex.l22_3 * G1_2 ;
G2_3 =  cnt->mex.l44_3 * G2_2 ;

// coefficient of fourth iteration

Gs_3   =  G1_3 + G2_3;
Vf1_3  = (Gs_0 * Vm_3  +  Gs_1 * Vm_2)  +  (Gs_2 * Vm_1  +
           Gs_3 * Vm_0);
Vm_4 =  (cnt->mex.l11_4 * Vm_3 + cnt->mex.l12_4 * G1_3) +
          (cnt->mex.l13_4 * G2_3 + cnt->mex.n12_4 * Vf1_3);
G1_4 =  cnt->mex.l22_4 * G1_3 ;
G2_4 =  cnt->mex.l44_4 * G2_3 ;

 // aggregate the partial solutions
Vm_k = ((Vm_0 + Vm_1) + (Vm_2 + Vm_3)) + Vm_4;
G1_k = ((G1_0 + G1_1) + (G1_2 + G1_3)) + G1_4;
G2_k = ((G2_0 + G2_1) + (G2_2 + G2_3)) + G2_4;

/* end solver equations PS4 */
```

Supplementary Figure 5: Core of the Parker-Sochacki HLS implementation. **(A)** pre-calculated constants, related to the underlying ODE system and solver. **(B)** declaration of the dynamic variables and coefficients of the Taylor-series, **(C)** unrolled PS-iterations of order 4.

A.

```
typedef struct

mex_tt {

float  c21;
Float  c22;

float   l11_1;
float   l12_1;
float   l13_1;
float   n12_1;
float   c11_1;
float   l22_1;
float   l44_1;


float   l11_2;
float   l12_2;
float   l13_2;
float   n12_2;
float   l22_2;
float   l44_2;


float   l11_4;
float   l12_4;
float   l13_4;
float   n12_4;
float   l22_4;
float   l44_4;


} mex_t;
```

B.

```
/* begin variables definition */

   float     Gxt,Gex,Gin,m1,m2;
   uint32_t  aer;
   union     {uint32_t i; float f;}  Vm, I1, I2;
   union     {uint32_t i; float f;}  Vm_nxt,I1_nxt,I2_nxt;
   uint96    ste,ste0,ste1,ste2;

   // method variables
   float  Vm_0, G1_0, G2_0;
   float  Vm_1, G1_1, G2_1,  Vf1_0;
   float  Vm_2, G1_2, G2_2, Vf1_1;
   float  Vm_3, G1_3, G2_3,  Vf1_2;
   float  Vm_4, G1_4, G2_4,  Vf1_3;
   float  Vm_k, G1_k, G2_k;
   /* end variables definition */
```

C.

```
/* begin solver equations RK4 */


m1   = Gxt + Gex + I1.f;
m2   = Gin      + I2.f;

Vm_0 = Vm.f;
G1_0 = m1;
G2_0 = m2;

// coefficient of first iteration

Vf1_0 =  (G1_0 + G2_0) * Vm_0;
Vm_1 = Vm_0 + (cnt->mex.l11_2 * Vm_0 + cnt->mex.l12_2 * G1_0) +
                     (cnt->mex.l13_2 * G2_0 + cnt->mex.n12_2 * Vf1_0) +
                      ( cnt->mex.c11_2);
G1_1 = G1_0 +  cnt->mex.l22_2 * G1_0 ;
G2_1 = G2_0 +  cnt->mex.l44_2 * G2_0;
```

C. (cont.)

```
// coefficient of second iteration

Vf1_1 =  (G1_1 + G2_1) * Vm_1;
Vm_2 = Vm_0 + (cnt->mex.l11_2 * Vm_1 + cnt->mex.l12_2 * G1_1) +
                     (cnt->mex.l13_2 * G2_1 + cnt->mex.n12_2 * Vf1_1) +
                     (cnt->mex.c11_2);
G1_2 = G1_0 +  cnt->mex.l22_2 * G1_1 ;
G2_2 = G2_0 +  cnt->mex.l44_2 * G2_1;

// coefficient of third iteration

Vf1_2 =  (G1_2 + G2_2) * Vm_2;
Vm_3 = Vm_0 + (cnt->mex.l11_1 * Vm_2 + cnt->mex.l12_1 * G1_2) +
                     (cnt->mex.l13_1 * G2_2 + cnt->mex.n12_1 * Vf1_2) +
                     (cnt->mex.c11_1);
G1_3 = G1_0 +  cnt->mex.l22_1 * G1_2 ;
G2_3 = G2_0 +  cnt->mex.l44_1 * G2_2;

// coefficient of fourth iteration

Vf1_3 =  (G1_3 + G2_3) * Vm_3;
Vm_4 = Vm_0 + (cnt->mex.l11_4 * Vm_3 + cnt->mex.l12_4 * G1_3) +
                     (cnt->mex.l13_4 * G2_3 + cnt->mex.n12_4 * Vf1_3) +
                     (cnt->mex.c11_4);
G1_4 = G1_0 +  cnt->mex.l22_4 * G1_3 ;
G2_4 = G2_0 +  cnt->mex.l44_4 * G2_3;

// aggregation

Vm_k = cnt->mex.c21*(Vm_2 + Vm_4) + cnt->mex.c22*(Vm_3 + Vm_1) ;
G1_k = cnt->mex.c21*(G1_2 + G1_4) + cnt->mex.c22*(G1_3 + G1_1);
G2_k = cnt->mex.c21*(G2_2 + G2_4) + cnt->mex.c22*(G2_3 + G2_1) ;

/* end solver equations RK4 */
```

Supplementary Figure 6: Core of the Runge-Kutta HLS implementation. **(A)** pre-calculated constants, related to the underlying ODE system and solver. **(B)** declaration of the dynamic variables and coefficients of the Taylor-series. **(C)** unrolled RK-iterations.

A.
```
#include <stdlib.h>
#include <stdio.h>
#include <stdint.h>
#include <math.h>
#include <ap_cint.h>
#define NSIZEPERPIPE 256
```

B.
```
#pragma HLS RESOURCE variable=cnt->mem.Sum core=RAM_T2P_BRAM
#pragma HLS RESOURCE variable=cnt->mem.Gextern core=RAM_1P_BRAM
#pragma HLS RESOURCE variable=cnt->mem.Gin     core=RAM_1P_BRAM
#pragma HLS RESOURCE variable=cnt->mem.Gex     core=RAM_1P_BRAM
#pragma HLS RESOURCE variable=cnt->mem.oFifo   core=RAM_1P_BRAM
```

C.
```
typedef struct prm_tt {
  float      Erest;     /* membrane V after spike   */
  float      vth;       /* membrane threshold       */
  uint16_t nSet;        /* number of active neurons */
} prm_t;
```

D.
```
typedef struct mem_tt {

  float        Gextern[NSIZEPERPIPE]; /* from poisson generator              */
  float        Gex[NSIZEPERPIPE];    /* excitatory lumped synapses          */
  float        Gin[NSIZEPERPIPE];    /* inhibitory lumped synapses          */
  uint96       Sum[NSIZEPERPIPE];    /* aggegated state variables ,3x32 b */
  uint32_t     AER[NSIZEPERPIPE];    /* absolute AER address                */
  uint32_t     oFifo[NSIZEPERPIPE]; /* ouput  buffer for detected spikes  */
  uint16_t     nOut; ;                /* number of valid entries  in oFifo   */
} mem_t;
```

E.
```
typedef struct cntnr_tt {
  mem_t      mem;
   prm_t     prm;
   mex_t     mex;
} cntnr_t;
```

F.
```
void ODE_SLV(cntnr_t *cnt) {
   uint16_t  neuronIndex, nOut;

    /* variables definition here */

   for (neuronIndex = 0 ; neuronIndex < cnt->prm.nSet ; neuronIndex++) {
#pragma HLS LOOP_TRIPCOUNT min=256 max=256
#pragma HLS PIPELINE II=1
#pragma AP DEPENDENCE variable=cnt->mem.Sum inter false
    nOut  = cnt->mem.nOut
    Gxt  = cnt->mem.Gextern[neuronIndex];
    Gex  = cnt->mem.Gex[neuronIndex];
    Gin  = cnt->mem.Gin[neuronIndex];
    aer  = cnt->mem.AER[neuronIndex];

    ste    = cnt->mem.Sum[neuronIndex];   /* extract full state             */
    ste0  = apint_get_range(ste,  31,   0);    /* Membrane voltage         */
    ste1  = apint_get_range(ste,  63,  32);  /*  excitatory conductance */
    ste2  = apint_get_range(ste,  95,  64); /*   inhibitory conductance */
    Vm.i   = (uint32_t) ste0;
    I1.i    = (uint32_t) ste1;
    I2.i    = (uint32_t) ste2;

    /* solver equations here */

    /* comparison with threshold, 0 refractory, update state                 */
    Vm_nxt.f           = (( Vm_k >= cnt->prm.vth) ) ? cnt->prm.Erest  : Vm_k;
    cnt->mem.nOut = (( Vm_k >= cnt->prm.vth) ) ? nOut + 1            : nOut;

    I1_nxt.f           = G1_k;
    I2_nxt.f           = G2_k;
    cnt->mem.oFifo[nOut] = aer;

    // collect all the bits and form an 96 bit update
    ste0 = (uint96) Vm_nxt.i;
    ste1 = apint_set_range(ste0,  63,  32, (uint96) I1_nxt.i);
    ste    = apint_set_range(ste1,  95,  64, (uint96) I2_nxt.i);
    cnt->mem.Sum[neuronIndex]  = ste;
  } // end for (neuronIndex = 0
}  // end ODE_SLV
```

Supplementary Figure 7: (A) include libraries and defines. **(B)** synthesis directives for memories: true-2-port and single-port block RAMs. **(C)** structure for representing neuron parameters. **(D)** structure including memories which store state variables and synaptic input. **(E)** structure comprising memories, neuron parameters, and pre-calculated method-specific coefficients. **(F)** top-level solver.

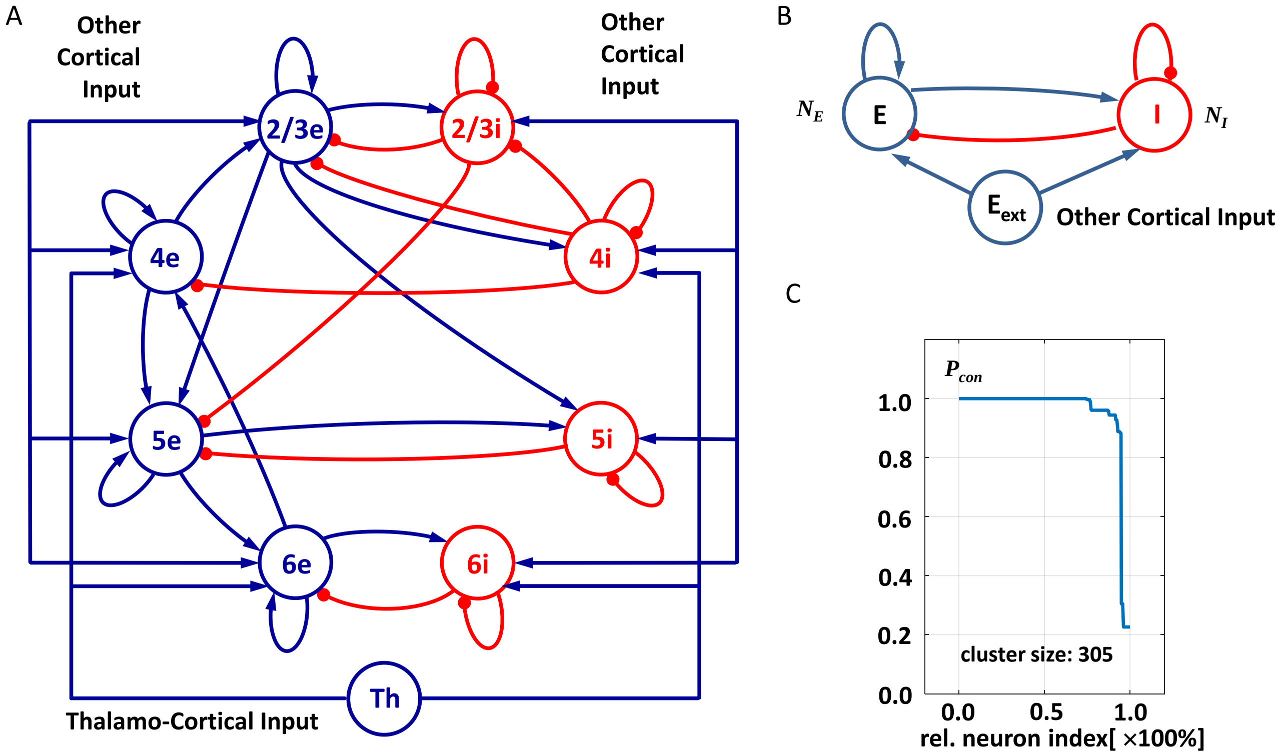

Supplementary Figure 8: Microcircuit model featuring 77169 LIF neurons **(A)**. $X \in$ {L2/3E, L2/3I, L4E, L4I, L5E, L5I, L6E, L6I} denotes a particular (unordered) population. Pointed arrowheads represent excitatory connections while round endpoints represent inhibitory connections. Illustration of a random network **(B)** after (Brunel, 2000). Connection probability of neurons to nodes in the microcircuit **(C)**.

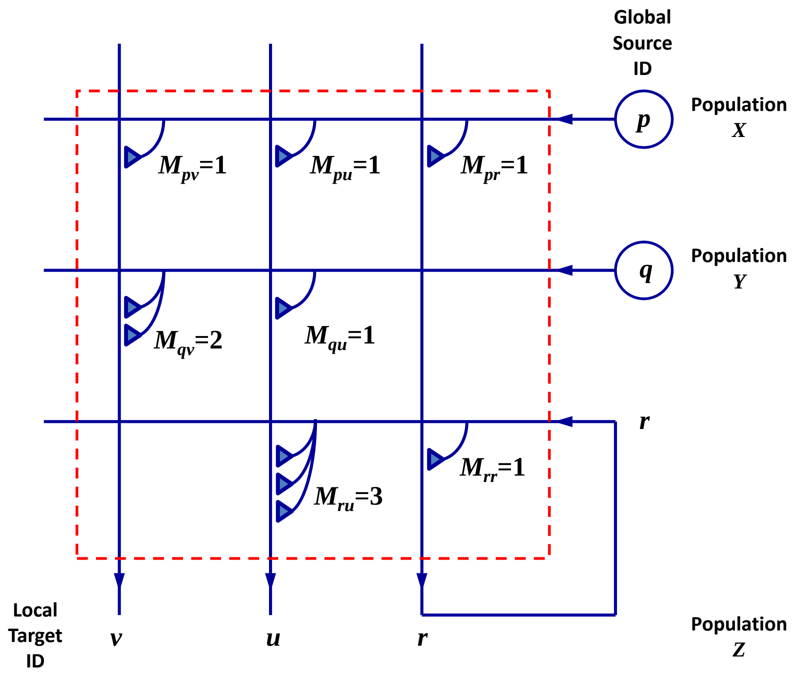

Supplementary Figure 9: Forms of synaptic connectivity. Individual pairs of neurons may be connected via a multiplicity of synapses (parameter *M*). Single connections are called *synapses*, connections with *M* >1 are called *multapes*, and synapses establishing self-connections are called *autapses* (e.g. connection r-r)

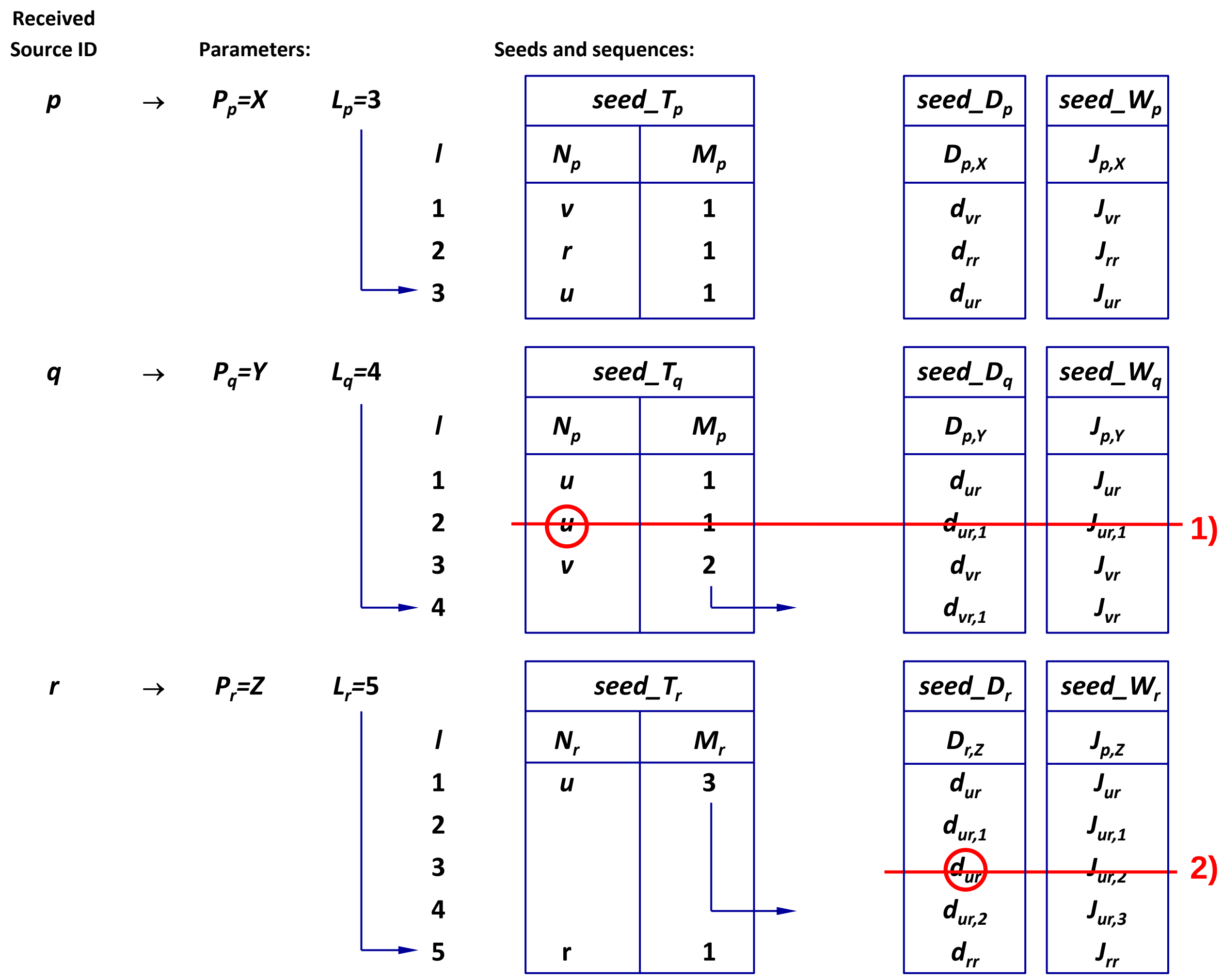

Supplementary Figure 10: Illustration of (N,M,D,J)-tupel sequences generated by the RTR unit representing the network in **Supplementary Figure 9**. The effect of incidentally generating twin numbers in discrete random sequences may result in 1) double-indexing of target neurons or 2) twin-delays.

2 Supplementary References

Butcher, J.C. (2016). *Numerical Methods for Ordinary Differential Equations*. Hoboken, NJ: Wiley. doi:10.1002/9781119121534

Davidson, A., Brüderle, D., Eppler, J., Kremkow, J., Muller, E., Pecevski, D., et al. (2008). PyNN: a Common Interface for neuronal Network Simulators. *Front. Neuroinform.* 2:11. doi: 10.3389/neuro.11.011.2008

Nordlie, E., Gewaltig, M.-O., Plesser, H.E. (2009). Towards Reproducible Descriptions of Neuronal Network Models. *PLoS Comput. Biol.*, 5:e1000456. doi: 10.1371/journal.pcbi.1000456

Parker, G. E. and Sochacki, J. S. (1996). Implementing the Picard iteration. *Neural, Parallel & Sci. Comput.*, 4, 97–112. Doi: 10.1155/s1687182004311058
